# Soluble CD13 is a potential mediator of neutrophil-induced thrombogenic inflammation in SARS-CoV-2 infection

**DOI:** 10.1172/jci.insight.184975

**Published:** 2025-04-01

**Authors:** Pei-Suen Tsou, Ramadan A. Ali, Chenyang Lu, Gautam Sule, Carmelo Carmona-Rivera, Serena Lucotti, Yuzo Ikari, Qi Wu, Phillip L. Campbell, Mikel Gurrea-Rubio, Kohei Maeda, Sharon E. Fox, William D. Brodie, Megan N. Mattichak, Caroline Foster, Ajay Tambralli, Srilakshmi Yalavarthi, M. Asif Amin, Katarina Kmetova, Bruna Mazetto Fonseca, Emily Chong, Yu Zuo, Michael D. Maile, Luisa Imberti, Arnaldo Caruso, Francesca Caccuri, Virginia Quaresima, Alessandra Sottini, Douglas B. Kuhns, Danielle Fink, Riccardo Castagnoli, Ottavia M. Delmonte, Heather Kenney, Yu Zhang, Mary Magliocco, Helen Su, Luigi Notarangelo, Rachel L. Zemans, Yang Mao-Draayer, Irina R. Matei, Mirella Salvatore, David Lyden, Yogendra Kanthi, Mariana J. Kaplan, Jason S. Knight, David A. Fox

**Affiliations:** 1 Division of Rheumatology, Department of Internal Medicine, and Clinical Autoimmunity Center of Excellence, University of Michigan, Ann Arbor, Michigan, USA.; 2 Division of Rheumatology, Department of Internal Medicine, the Third Affiliated Hospital, Sun Yat-Sen University, Guangzhou, China.; 3 Systemic Autoimmunity Branch, National Institute of Arthritis and Musculoskeletal and Skin Diseases, NIH, Bethesda, Maryland, USA.; 4 Department of Pediatrics, Weill Cornell Medical College, New York, New York, USA.; 5 Department of Pathology, Louisiana State University, Health Sciences Center, New Orleans, Louisiana, USA.; 6 School of Medical Science, University of Campinas (UNICAMP), Campinas, Brazil.; 7 Department of Anesthesiology, University of Michigan, Ann Arbor, Michigan, USA.; 8 Section of Microbiology, University of Brescia, Brescia, Italy.; 9 Clinical Chemistry Laboratory, ASST Spedali Civili of Brescia, Brescia, Italy.; 10 Leidos Biomedical Research, Inc.; Frederick National Laboratory for Cancer Research, Frederick, Maryland, USA.; 11 Division of Intramural Research, National Institute of Allergy and Infectious Diseases, NIH, Bethesda, Maryland, USA.; 12 Division of Pulmonary & Critical Care Medicine, Department of Internal Medicine; and Program in Cellular and Molecular Biology, School of Medicine, University of Michigan, Ann Arbor, Michigan, USA.; 13 Multiple Sclerosis Center of Excellence, Arthritis and Clinical Immunology Research Program, Oklahoma Medical Research Foundation (OMRF), Oklahoma City, Oklahoma, USA.; 14 Joan and Sanford I. Weill Department of Medicine and Department of Population Health Sciences, Weill Cornell Medical College, New York, New York, USA.; 15 Division of Intramural Research, National Heart, Lung, and Blood Institute, NIH, Bethesda, Maryland, USA.

**Keywords:** COVID-19, Immunology, Infectious disease, Endothelial cells, Neutrophils, Thrombosis

## Abstract

The soluble variant of the ectopeptidase CD13 (sCD13), released from the cell surface by matrix metalloproteinase 14 (MMP14), is a potent pro-inflammatory mediator, displaying chemotactic, angiogenic, and arthritogenic properties through bradykinin receptor B1 (B1R). We revealed a link between sCD13 and amplified neutrophil-mediated inflammatory responses in SARS-CoV-2 infection. sCD13 was markedly elevated in patients with COVID-19 and correlated with disease severity and variants, ethnicity, inflammation markers, and neutrophil extracellular trap formation (NETosis). Neutrophils treated with sCD13 showed heightened NETosis and chemotaxis, which were inhibited by sCD13 receptor blockade. Meanwhile sCD13 did not induce platelet aggregation. Single-cell analysis of COVID-19 lungs revealed coexpression of CD13 and MMP14 by various cell types, and higher CD13 expression compared with controls. Neutrophils with high CD13 mRNA were enriched for genes associated with immaturity, though CD13 protein expression was lower. Histological examination of COVID-19 lungs revealed CD13-positive leukocytes trapped in vessels with fibrin thrombi. Flow cytometry verified the presence of B1R and a second sCD13 receptor, protease-activated receptor 4, on monocytes and neutrophils. These findings identify sCD13 as a potential instigator of COVID-19–associated NETosis, potentiating vascular stress and thromboembolic complications. The potent pro-inflammatory effects of sCD13 may contribute to severe COVID-19, suggesting that sCD13 and its receptors might be therapeutic targets.

## Abstract 2

Thw inflammatory mediator soluble CD13 (sCD13) is markedly elevated in serum and lungs of patients with SARS-CoV-2 , and is a trigger of immunothrombosis in these patients.

## Graphical abstract

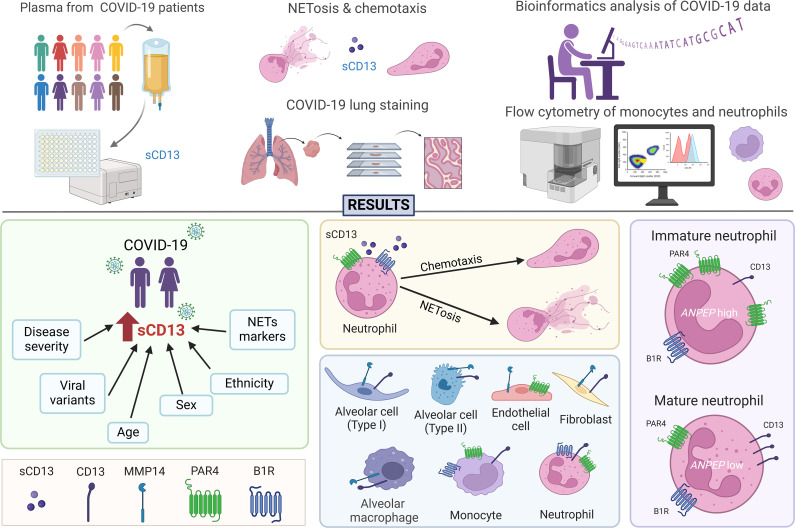

## Introduction

Since March 11, 2020, the day the World Health Organization declared COVID-19 a pandemic, this disease has resulted in clinical, social, and economic disruption; infected more than 775 million individuals worldwide; and claimed the lives of more than 1.1 million people in the United States. Caused by SARS-CoV-2, this disease is highly transmissible and has a higher mortality rate during hospitalization than influenza (1). The initial symptoms of COVID-19 are similar to other viral diseases, such as fever, headache, muscle pain, fatigue, and intestinal symptoms (2). In certain cases the disease progresses to acute respiratory distress syndrome and organ failure. Evidence also indicates that various COVID-19 symptoms may persist even after the resolution of the acute infection, a condition now referred to as long COVID. The severity of COVID-19 appears to be dependent on genetics, age/sex/ethnicity, abnormal coagulation and thrombosis, immunological dysregulation, and underlying risk factors and comorbidities (3–7). Deficiency in type I and type III interferons, caused by neutralizing antibodies or defects in signaling, and low induction of local and systemic interferon (IFN) responses, appear to be factors in the severity of SARS-CoV-2 infection (8–10). Cells involved in innate immunity, including mast cells and neutrophils, also contribute to SARS-CoV-2 severity (11, 12). It has been shown that neutrophils, by releasing neutrophil extracellular traps (NETs), contribute to the formation of clots in this disease (13, 14). Here we propose a mechanism involving a critical role for the soluble form of aminopeptidase N (CD13, EC 3.4.11.2) in the pathogenesis of neutrophil-mediated thromboinflammation in COVID-19.

CD13 is an ectopeptidase that is highly expressed on stromal and myeloid cells in joints, lung, and other tissues (15). Recent work has revealed a surprising range and potency of inflammatory effects of the soluble form of CD13 (sCD13), which are independent of its enzymatic activity (15). We have previously shown that sCD13, which is cleaved from its membrane-bound form by matrix metalloproteinase 14 (MMP14), activates endothelial cells (ECs), monocytes, and T cells, as well as rheumatoid arthritis (RA) fibroblast-like synoviocytes and dermal fibroblasts (16–19). Modulating the functions of these various cell types, sCD13 is a potent pro-inflammatory mediator with strong chemotactic, angiogenic, pro-fibrotic, and arthritogenic properties. The source of sCD13 could arise from cell types that coexpress MMP14 and CD13 or from CD13-expressing cells that are in close proximity to MMP14-expressing cells.

We recently demonstrated that sCD13 elicits its pro-inflammatory effects through the bradykinin receptor B1 (B1R) (19, 20). In screening of a G-coupled receptor library, protease-activated receptor-4 (PAR4) was also identified as a second potential receptor for sCD13. Interestingly, membrane-bound CD13 is a known receptor for coronavirus HCoV-229E (21, 22). However, the enzymatic activity is not critical for viral entry, since inhibition of its peptidase function does not block binding of HCoV-229E or infection (21).

NETs are webs of chromatin and antimicrobial proteins released by neutrophils via a cell death program known as NETosis (23). NETs are known to trigger formation of thrombi in antiphospholipid syndrome (24) and likely in other inflammatory and rheumatologic conditions. NET-associated markers have been shown to correlate with disease severity in patients with COVID-19 (14, 25). Moreover, sera from patients with COVID-19 trigger healthy neutrophils to undergo NETosis (25). Follow-up studies further showed that NET-associated markers in patient sera, including citrullinated histone H3 (Cit-H3), cell-free DNA, myeloperoxidase-DNA complexes (MPO-DNA), and calprotectin, were associated with higher risk of thrombotic events in patients with COVID-19 (26). Given the potent pro-inflammatory effect of sCD13 on various immune cells, we hypothesized that sCD13 triggers the aggressive inflammatory responses that characterize severe COVID-19, possibly including NETosis. Here, we analyzed sCD13 in blood samples from patients with COVID-19 from 3 cohorts and determined the effect of sCD13 on neutrophil function. We also queried published single-cell RNA-Seq datasets established using COVID-19 lungs, nasopharyngeal swabs, and peripheral immune cells to assess the potential contributions of various cell types pertinent to lung inflammation to the production of sCD13. The results suggest that the powerful pro-inflammatory effects of sCD13, as well as CD13 in its membrane-bound form, may account for important components of the unusual hyperinflammatory complications of SARS-CoV-2 infection.

## Results

### Increased serum sCD13 levels in patients with COVID-19.

**Figure 1:**
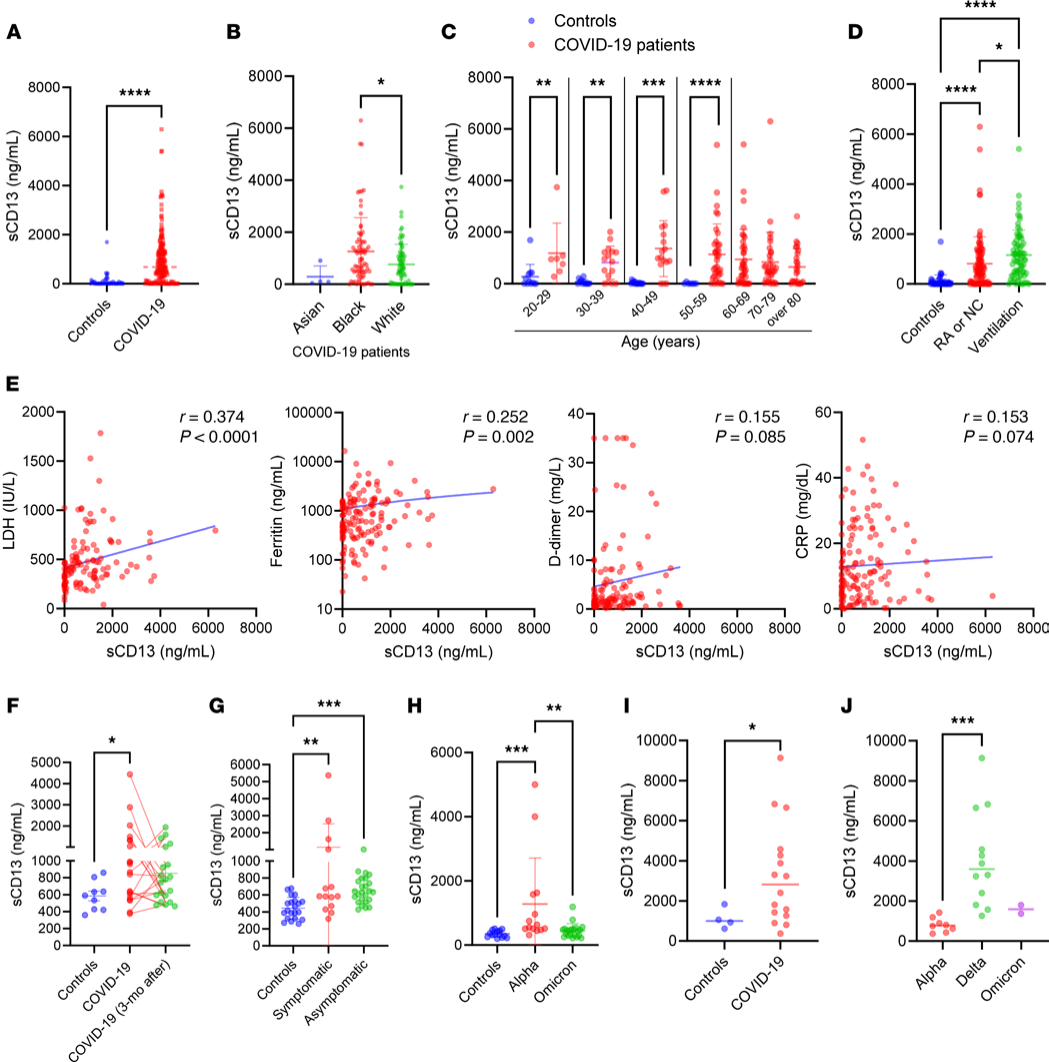
Circulating sCD13 levels are elevated in patients with COVID-19 and correlate with inflammatory markers. (**A**) Compared with healthy controls, significant elevation of sCD13 was observed in patients with COVID-19 (controls *n* = 48, COVID-19 *n* = 172). (**B**) Black patients showed significantly higher levels of sCD13 compared with White patients with COVID-19 (Asian *n* = 4, Black *n* = 77, White *n* = 73). (**C**) Significantly higher sCD13 levels were observed in patients with COVID-19 at each age group compared with healthy controls (controls *n* = 48, COVID-19 *n* = 172). (**D**) Patients requiring mechanical ventilation had significantly higher levels of sCD13 compared with patients breathing room air (RA) or with nasal cannula (NC) (controls *n* = 46, RA or NC *n* = 95, ventilation *n* = 72). (**E**) Significant positive correlation between sCD13 levels and clinical labs ferritin (*n* = 144) or lactate dehydrogenase (LDH, *n* = 118) was observed. D-dimer (*n* = 124) and C-reactive protein (CRP, *n* = 138) demonstrated positive slopes that were not statistically significant. (**F**) In a separate cohort, sCD13 was significantly elevated in patients with COVID-19 (*n* = 20) compared with healthy controls (*n* = 9), while levels did not significantly change 3 months after the diagnosis (*n* = 20). (**G**) Both symptomatic (*n* = 14) and asymptomatic patients (*n* = 25) showed significantly higher sCD13 levels compared with healthy controls (*n* = 21). (**H**) sCD13 levels were significantly higher in COVID-19 patients with the Alpha variant (*n* = 14) compared with healthy controls (*n* = 14) and patients with the Omicron variant (*n* = 21). (**I**) Significant elevation of sCD13 in patients with COVID-19 (*n* = 16) was also observed in a third cohort compared with healthy controls who were not vaccinated (*n* = 4). (**J**) Significant elevation of sCD13 was observed in COVID-19 patients with the Delta variant (*n* = 12) compared with patients with the Alpha (*n* = 8) or Omicron variants (*n* = 2). Results are expressed as mean ± SD. *P < 0.05, **P < 0.01, ***P < 0.001, ****P < 0.0001. Significance was determined by Mann-Whitney test (**A** and **C**), Kruskal-Wallis test (**B**, **D**, **F**–**H**, and **J**), Spearman’s test (**E**), and unpaired, 2-tailed Student’s *t* test (**I**).

We observed significant elevation of sCD13 in a cohort of hospitalized patients with COVID-19 compared with healthy controls (Figure 1A), and the mean was 10-fold higher in patients with COVID-19 versus healthy controls, *P* < 0.0001). In patients with COVID-19, serum sCD13 levels were affected by patient race and age (Figure 1, B and C). There were no significant differences in sCD13 levels between male (*n* = 97, 1,099 ± 1,161 ng/mL) and female patients (*n* = 76, 770 ± 899 ng/mL, *P* = 0.06, Supplemental Figure 1A; supplemental material available online with this article; https://doi.org/10.1172/jci.insight.184975DS1). While sCD13 levels mostly did not differ based on patient comorbidities (“All” in Supplemental Tables 1 and 2), male patients with diabetes had significantly higher levels of sCD13 compared with female patients with diabetes (Supplemental Table 1, *P* = 0.001). Black patients (*n* = 77) showed significantly higher levels of sCD13 when compared with White patients (*n* = 73, *P* < 0.05, Figure 1B). When comorbidities were analyzed with race, we found that Black patients with cancer, hypertension, or a history of smoking had significantly higher levels of sCD13 compared with White patients (Supplemental Table 2, *P* < 0.05). Significantly higher sCD13 levels were observed in patients with COVID-19 compared with healthy controls in each age group (20–59 years old, Figure 1C). In addition, a negative correlation between age and sCD13 levels was observed; this inverse relationship was more significant in healthy controls (*r* = –0.397, *P* = 0.006) than in patients with COVID-19 (*r* = –0.176, *P* = 0.02, Supplemental Figure 2). To further explore the role of sCD13 in patients with COVID-19, we determined the relationship between sCD13 levels and other disease parameters. When hospitalized patients were stratified based on their clinical status, patients requiring mechanical ventilation (*n* = 72) had significantly higher sCD13 levels compared with those with milder COVID-19 symptoms who received oxygen by nasal cannula or were on room air (*n* = 95, Figure 1D). However, there were no differences in sCD13 levels when the outcomes (death, discharged, or unknown) of the patients were compared (Supplemental Figure 3).

We next analyzed the association between sCD13 levels and inflammatory markers (Figure 1E). We observed a strong correlation between sCD13 and LDH (*r* = 0.374, *P* < 0.0001). A significant association was also found between sCD13 and ferritin (*r* = 0.252, *P* = 0.002). D-dimer and CRP demonstrated positive slopes that were not statistically significant (*P* = 0.085 and *P* = 0.074, respectively). Interestingly, the association between sCD13 and LDH or ferritin was more significant in men than in women (Supplemental Figure 1B). In addition, serum levels of sCD13 were more strongly correlated with LDH in Black patients (*r* = 0.450, *P* = 0.0008) compared with White patients (*r* = 0.364, *P* = 0.01, Supplemental Figure 4A).

To validate our findings, we included 2 additional cohorts. In patients from an Italian cohort (27), serum sCD13 was significantly elevated compared with healthy controls and remained elevated 3 months after COVID-19 diagnosis (Figure 1F). In addition, sCD13 was significantly elevated in both symptomatic and asymptomatic patients with COVID-19 (Figure 1G). In this cohort, plasma levels of sCD13 could be compared in patients infected with Alpha and Omicron variants of SARS-CoV-2. We found that sCD13 was significantly elevated in patients with the Alpha variant compared with healthy controls, while patients infected with the Omicron variant showed significantly lower sCD13 levels compared with patients with the Alpha variant (Figure 1H). Among the patients infected with the Omicron variant, the levels of sCD13 correlated strongly with many pro-inflammatory cytokines, including IL-2, IL-16, MCP-4, IL-8, VEGF, and MIP-1β (Supplemental Figure 5). In the third cohort, similar results were found, showing that plasma sCD13 levels were significantly elevated in patients with COVID-19 compared with healthy controls (Figure 1I). In addition, patients with the Delta variant had significantly higher levels of sCD13 compared with those infected with the Alpha or Omicron variants (Figure 1J). The differences between the cohorts observed in the levels of sCD13 in Figure 1, H and J, may be multifactorial, involving differences in viral strains, host factors, and sample processing procedures.

### Levels of sCD13 correlate with NETosis-associated markers in patients with COVID-19.

**Figure 2:**
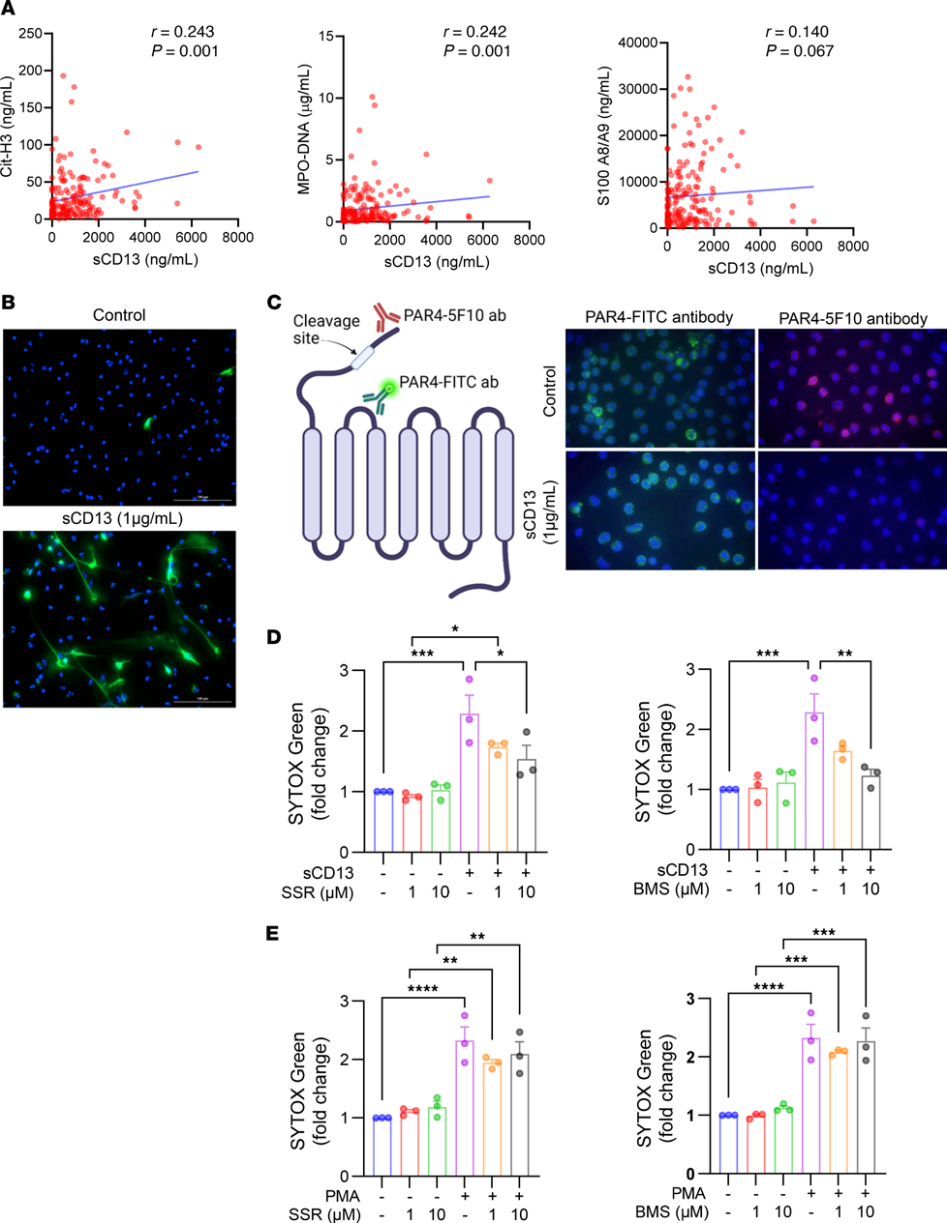
sCD13 correlates with NETosis-associated markers in patients with COVID-19 and activates neutrophils. (**A**) sCD13 showed significant correlations with Cit-H3 (*n* = 172) and MPO-DNA (*n* = 172) in patients with COVID-19 while S100 A8/A9 (*n* = 172) showed a positive slope but not a statistically significant correlation. (**B**) Representative images of neutrophils isolated from peripheral blood and analyzed after stimulation with PBS or sCD13. Panels show merged images of NETs in which neutrophil elastase was stained green by immunofluorescence and DNA was stained blue by Hoechst 33342. *n* = 3 technical replicates. Scale bar: 100 μm. (**C**) sCD13 blocked the staining of PAR4-5F10 antibodies, which recognize only the inactivated/uncleaved form of PAR4 yet had no effect on PAR4-FITC antibodies, which recognize both the activated and inactivated forms. *n* = 3 technical replicates, repeated 2 times. Original magnification, ×1,000. (**D**) sCD13-induced NETosis was blocked by B1R inhibitor SSR-240612 and PAR4 inhibitor BMS-986120 (all *n* = 3). (**E**) PMA-induced NETosis was not impacted by the B1R or PAR4 inhibitors (all *n* = 3). Results are expressed as mean ± SD. *P < 0.05, **P < 0.01, ***P < 0.001, ****P < 0.0001. Significance was determined by Spearman’s test (**A**) and 1-way ANOVA (**D** and **E**).

As elevated NETosis markers have been detected in patients with COVID-19 and are associated with higher risk of thrombosis (25, 26), we assessed whether sCD13 levels correlate with markers of NETosis. We detected a strong positive correlation with Cit-H3 (*r* = 0.243, *P* = 0.001) and MPO-DNA complexes (*r* = 0.242, *P* = 0.001, Figure 2A), which are markers regarded as specific for NET remnants. S100A8/A9 (calprotectin), a neutrophil activation marker, demonstrated a positive slope that was not statistically significant (*r* = 0.140, *P* = 0.067, Figure 2A). Similar to what was observed with inflammatory markers, sCD13 levels in men showed stronger correlation with NETosis-associated markers (Supplemental Figure 1C). However, when race was considered, White patients showed significantly stronger associations for sCD13 and NETosis markers (Supplemental Figure 4B). We also examined the levels of sCD13 in patients with sepsis, who also have inflammatory thrombosis. We found that sCD13 was significantly elevated in patients with sepsis compared with healthy controls (Supplemental Figure 6A). However, unlike what we discovered in COVID-19, sCD13 was not significantly correlated with Cit-H3 (Supplemental Figure 6B). The sCD13 levels in patients with sepsis also did not correlate with S100A8/A9.

### sCD13 activates neutrophils.

To examine whether sCD13 activates neutrophils, we performed functional assays, including NETosis and chemotaxis, using neutrophils isolated from healthy controls. By immunofluorescence microscopy, we demonstrated NET release by staining extracellular chromatin structures with neutrophil elastase (Figure 2B). We also performed 2 additional assays, release of DNA-bound MPO enzyme and externalization of DNA, to measure NETosis and showed that sCD13 induced NETosis significantly compared with controls (Supplemental Figure 7A). To verify that sCD13 cleaves and activates PAR4 in neutrophils, we employed 2 PAR4 antibodies: PAR4-5F10, which recognizes the uncleaved form, and PAR4-FITC, which binds to the extracellular loop and detects both the cleaved and uncleaved forms (Figure 2C). Pretreatment of neutrophils with sCD13 resulted in reduced staining with the PAR4-5F10 antibody, but not with the PAR4-FITC antibody, indicating that sCD13 cleaves off the signaling peptide at the N-terminal of PAR4. To determine the involvement of the 2 receptors for sCD13, B1R and PAR4, in NETosis, we incorporated B1R inhibitor SSR-240612 or PAR4 inhibitor BMS-986120 in the NETosis assay and found that both inhibitors blocked sCD13-induced NETosis (Figure 2D). In contrast, PMA-induced NETosis was not affected by B1R or PAR4 blockade (Figure 2E). We also found that there was an additive effect in blocking sCD13-induced NETosis when both inhibitors were combined at lower doses (1 μM, Supplemental Figure 7B). In addition to NETosis, we demonstrated that sCD13 dose-dependently induced neutrophil chemotaxis in a modified Boyden chamber, with concentrations of 1 and 2 μg/mL reaching statistical significance compared with phosphate buffered saline (PBS) control (Supplemental Figure 7C). In a separate experiment, neutrophil chemotaxis was measured in an Incucyte system (Sartorius), and similar results were obtained; sCD13 induced neutrophil chemotaxis, which was consistently blocked by the PAR4 inhibitor (Supplemental Figure 7D). These results suggest that sCD13 activates neutrophils through its receptors, B1R and PAR4.

Given that NETosis can activate thrombus formation and sCD13 induces NET formation, we next investigated whether sCD13 has a direct effect on platelets. Unlike a PAR4 agonist or thrombin, sCD13 did not significantly induce platelet aggregation (Supplemental Figure 7E). Furthermore, thrombin failed to induce NETosis in neutrophils isolated from healthy controls (Supplemental Figure 7F). These findings suggest that sCD13 promotes thrombosis through a mechanism involving NETosis, rather than through a direct effect on platelets, distinguishing its action from that of thrombin.

### Cells expressing CD13 are present in lungs, and CD13 expression is increased in the lungs of patients with COVID-19.

**Figure 3:**
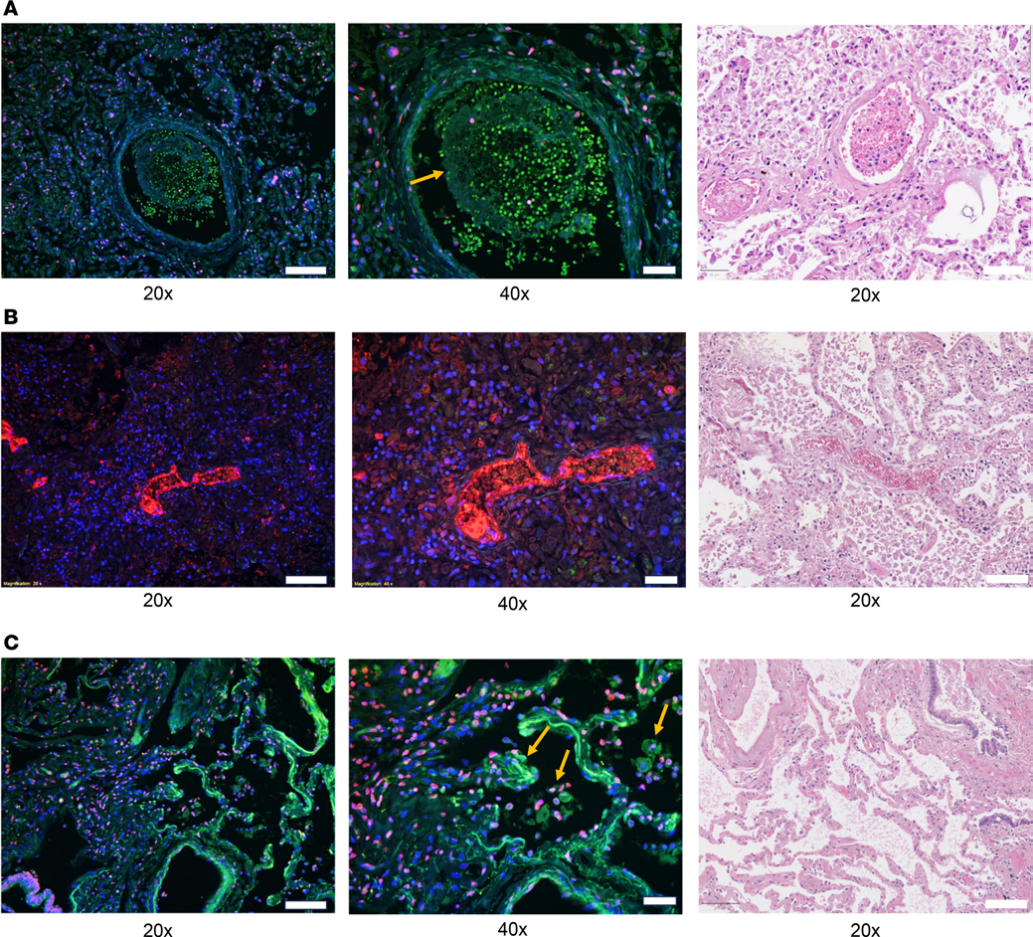
CD13 is highly expressed in lungs from patients with COVID-19. Immunofluorescence and H&E staining were performed on lung tissues obtained from autopsies of patients with cause of death attributed to COVID-19, targeting CD13, Cit-H3, and nuclear staining DAPI. (**A**) A pulmonary blood vessel containing a fibrin thrombus (arrow) with numerous entrapped leukocytes with positivity for CD13 (green). These cells can also be seen adherent to the vessel wall. Rare cells with histone H3 labeling (magenta) are interspersed. (**B**) Small pulmonary blood vessel containing a blood clot with extensive CD13 labeling (red), particularly in areas adjacent to the endothelial lining of the vessel. (**C**) Alveolar spaces are lined by capillaries. Desquamated pneumocytes and alveolar macrophages can be seen within the spaces (arrows), along with cells positive for histone H3 (magenta). Histone H3–labeled cells can also be seen along the endothelial lining and within the adjacent interstitium. Occasional cells with CD13 positivity (green) can also be seen along the alveolar endothelial lining. Representative lung tissue images of 3 patients with COVID-19 are shown. Original magnification, ×200–×400. Scale bar: 20×: 50 μm; 40×: 20 μm.

To gain more insights into the impact of sCD13 on neutrophils in patients with COVID-19, we performed tissue staining of autopsy lungs from patients with COVID-19 (Figure 3). We observed CD13-positive leukocytes entrapped in pulmonary blood vessels containing fibrin thrombi (Figure 3A). Similar results were observed in another patient, showing extensive CD13 labeling in a small pulmonary blood vessel containing a blood clot, particularly in areas adjacent to the endothelial lining (Figure 3B). Figure 3C shows alveolar spaces lined by capillaries. Desquamated pneumocytes and alveolar macrophages can be seen within the spaces, along with cells positive for Cit-H3, which can also be seen along the endothelial lining, and within the adjacent interstitium. Similar results were observed using neutrophil elastase as a marker for neutrophils (Supplemental Figure 8). These results show that CD13 is expressed highly in COVID-19 lungs, though CD13 and Cit-H3 do not appear to tightly colocalize with each other. It is possible that these CD13-expressing cells are the source of sCD13 in the lung, which could attract neutrophils and other leukocytes to localize in the tissue.

**Figure 4:**
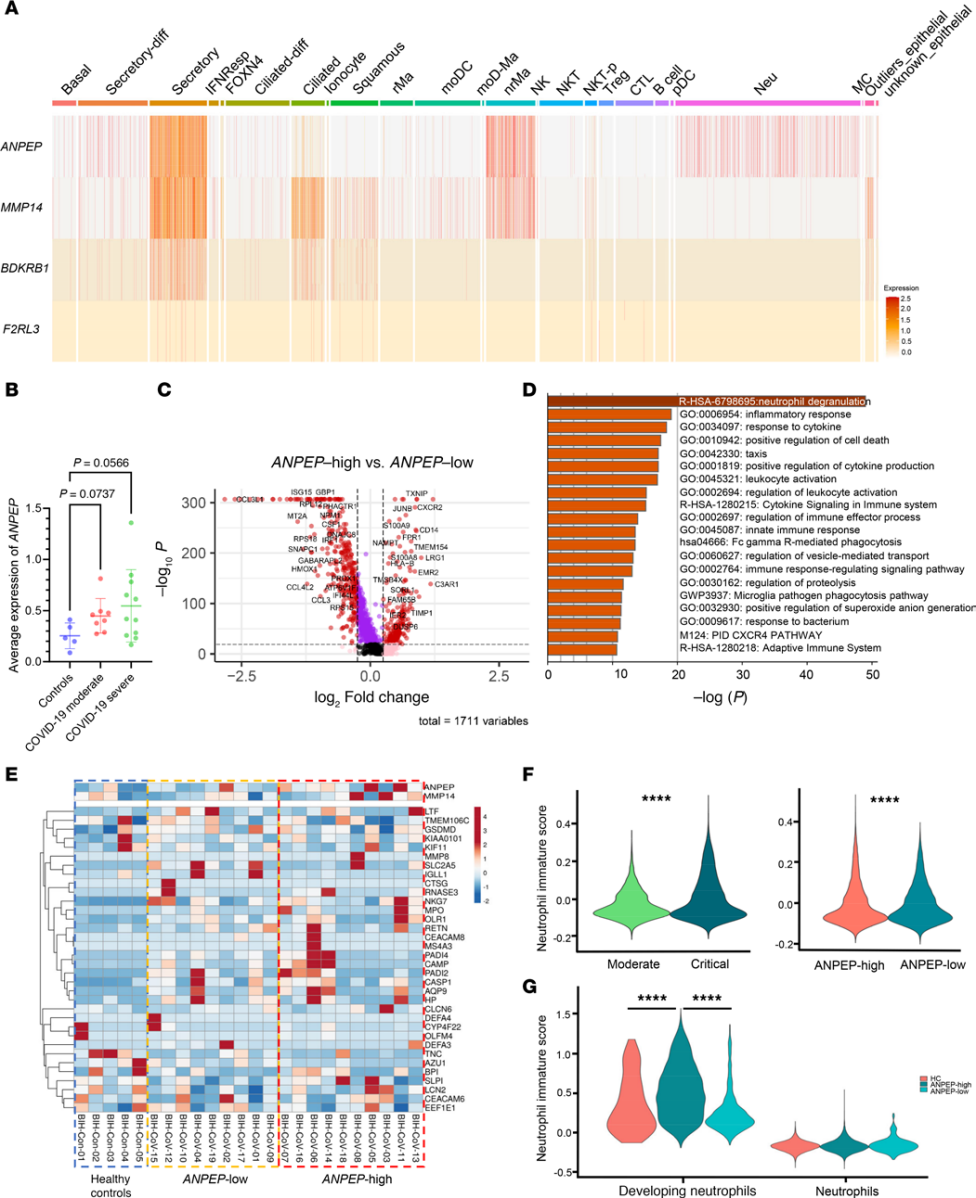
Cells expressing CD13, MMP14, B1R, and PAR4 are present in lungs in patients with COVID-19, and cellular levels of CD13 mRNA correlate with disease severity and neutrophil maturity. Single-cell RNA-Seq results of nasopharyngeal/pharyngeal swabs, bronchial brushings and bronchial lavages were generated from patients with COVID-19 (*n* = 19) and healthy controls (*n* = 5). Data are extracted from Chua et al. (29). (**A**) Both *ANPEP* (codes for CD13) and *MMP14* are expressed on various epithelial cells and macrophages. *ANPEP* is also expressed on neutrophils while *MMP14* is expressed on mast cells. *BDKRB1* (codes for B1R) is expressed on some epithelial cells with the highest expression on secretory cells and ciliated cells. *F2RL3* (codes for PAR4) was barely detected in this dataset but seemed to be expressed by epithelial cells. Cell abbreviations are defined in Supplemental Figure 8. (**B**) Cellular *ANPEP* expression in nasopharyngeal cells is elevated in patients with COVID-19 (moderate COVID-19 *n* = 8, severe COVID-19 *n* = 11) compared with healthy controls (*n* = 5). (**C**) Using the median expression levels of *ANPEP* in patients, patients with COVID-19 were divided into 2 populations: *ANPEP*-high and *ANPEP*-low. Differentially expressed genes in neutrophils from these 2 groups were shown in the volcano plot (*P* < 1 × 10^–20^, |log_2_(fold-change)| < 0.25). (**D**) Pathway analysis of the differentially expressed genes in *ANPEP*-high and *ANPEP*-low neutrophils showed pathways related to neutrophil degranulation, leukocyte activation, and inflammation. (**E**) The neutrophils from *ANPEP*-high patients with COVID-19 (*n* = 10) showed a gene signature of immature-like neutrophils characterized by the overexpression of genes coding for several granule-content proteins (healthy controls *n* = 5, *ANPEP*-low *n* = 9). (**F**) The score for neutrophil immaturity was higher in critically ill patients with COVID-19 (*n* = 11) compared with moderate patients (left, *n* = 8). The median neutrophil-immaturity score of neutrophils in *ANPEP*-high patients (*n* = 10) was lower than that in *ANPEP*-low patients (right, *n* = 9). (**G**) The neutrophil-immaturity signature was most prominent in *ANPEP*-high patients (*n* = 10) as developing neutrophils versus mature neutrophils from the peripheral blood were examined (healthy controls *n* = 5, *ANPEP*-low *n* = 9). Results are expressed as mean ± SD. ****P < 0.0001. Significance was determined by 1-way ANOVA (**B** and **G**) and Mann-Whitney test (**F**).

To further determine the potential cellular sources of CD13 in the lung, we extracted publicly available single-cell RNA-Seq datasets that were generated using tissues or blood from patients with COVID-19 (28–30). We first examined the expression pattern of CD13 (*ANPEP*), its sheddase MMP14, and the 2 receptors B1R (*BDKRB1*) and PAR4 (*F2RL3*). As shown in Figure 4A and Supplemental Figure 9, we analyzed single-cell RNA-Seq data from airway specimens (including nasopharyngeal tissue, bronchial tissue, and bronchial lavage fluid) procured from patients with COVID-19 (29). *ANPEP* was expressed by ciliated cells, secretory cells, macrophages, and neutrophils. *MMP14* was expressed in a similar pattern as *ANPEP* in various epithelial cells, while it was highly expressed in dendritic cells, macrophages, and mast cells. *BDKRB1* was expressed mainly on epithelial cells, while *F2RL3* had very low expression levels in this dataset. Although CD13 is abundantly expressed on neutrophils with low *MMP14* levels, these cells could still be possible sources of sCD13 when they are in close contact with MMP14-expressing cells. Similar observations were found in another dataset generated in lung tissues from patients who died with COVID-19 and underwent rapid autopsy (28). Both *ANPEP* and *MMP14* were expressed on various epithelial cells, fibroblasts, NK/T cells, and macrophages/monocytes (Supplemental Figure 10). With a larger sample size, *F2RL3* was detectable in this dataset and predominantly expressed on endothelial cells (ECs), neuronal cells, and smooth muscle cells. *BDKRB1* was expressed on some epithelial cells and fibroblasts. Since we previously showed that MMP14 cleaves CD13 from cell membranes of tissue stromal cells (synovial fibroblasts) to generate the soluble form (16), these results suggests that cells in the lung are able to shed sCD13, which in turn can act on receptors B1R or PAR4 to exert its effects.

Since *ANPEP* was highly expressed on various cell types in the lung, we then determined whether cellular *ANPEP* expression levels were altered in patients with COVID-19. Indeed, *ANPEP* was upregulated in cells collected from the airway specimens of patients with COVID-19 compared with healthy controls, specifically in patients with moderate disease (Figure 4B). In light of the importance of neutrophil dysfunction in the pathophysiology of COVID-19, especially in severe cases, we analyzed the expression level of neutrophil *ANPEP* in patients with COVID-19 who were divided into 2 populations, *ANPEP*-high (above the median) versus *ANPEP*-low (below the median). Interestingly, many pro-inflammatory genes including *S100A8* and *S100A9*, chemotaxis genes such as *CXCR2* and *FPR1*, and NETosis-associated genes such as *TIMP1* were highly expressed in neutrophils of *ANPEP*-high patients with COVID-19 compared with *ANPEP*-low patients with COVID-19 (Figure 4C). Pathway analysis of the differentially expressed genes in neutrophils from these 2 populations showed pathways related to neutrophil degranulation, leukocyte activation, and inflammation (Figure 4D). In addition, the neutrophils from *ANPEP*-high patients with COVID-19 showed a gene signature of immature-like neutrophils characterized by the overexpression of genes encoding several proteases that are contained in granules (31) (Figure 4E). The score for neutrophil immaturity was higher in critically ill patients with COVID-19 compared with moderate patients with COVID-19 (Figure 4F). Although the neutrophil-immaturity score in *ANPEP*-high patients with COVID-19 was highly variable, the median neutrophil-immaturity score of neutrophils in *ANPEP*-high patients with COVID-19 was higher than that in *ANPEP*-low patients with COVID-19 (Figure 4F). To further validate our findings, we analyzed an additional single-cell RNA-Seq dataset generated from peripheral immune cells from healthy controls and patients with COVID-19 (30). We found similar results, showing that COVID-19 patients with higher *ANPEP* expression levels in immune cells had more severe disease based on the WHO severity score for COVID-19 (Supplemental Figure 11A). In addition, differentially expressed genes in neutrophils from *ANPEP*-high versus *ANPEP*-low patients with COVID-19 were enriched in pathways involved in neutrophil function and inflammation (Supplemental Figure 11, B and C). Neutrophils from *ANPEP*-high patients with COVID-19 also showed a gene signature of immature neutrophils (Supplemental Figure 11D). The neutrophil immaturity signature was most prominent in *ANPEP*-high patients with COVID-19 as we examined developing neutrophils versus mature neutrophils from the peripheral blood as depicted in Figure 4G. Based on these results, in addition to its soluble form, cellular CD13 expression potentially plays a role in neutrophil function and maturity.

### CD13 and the receptors for its soluble form are expressed and inducible on neutrophils and monocytes.

**Figure 5:**
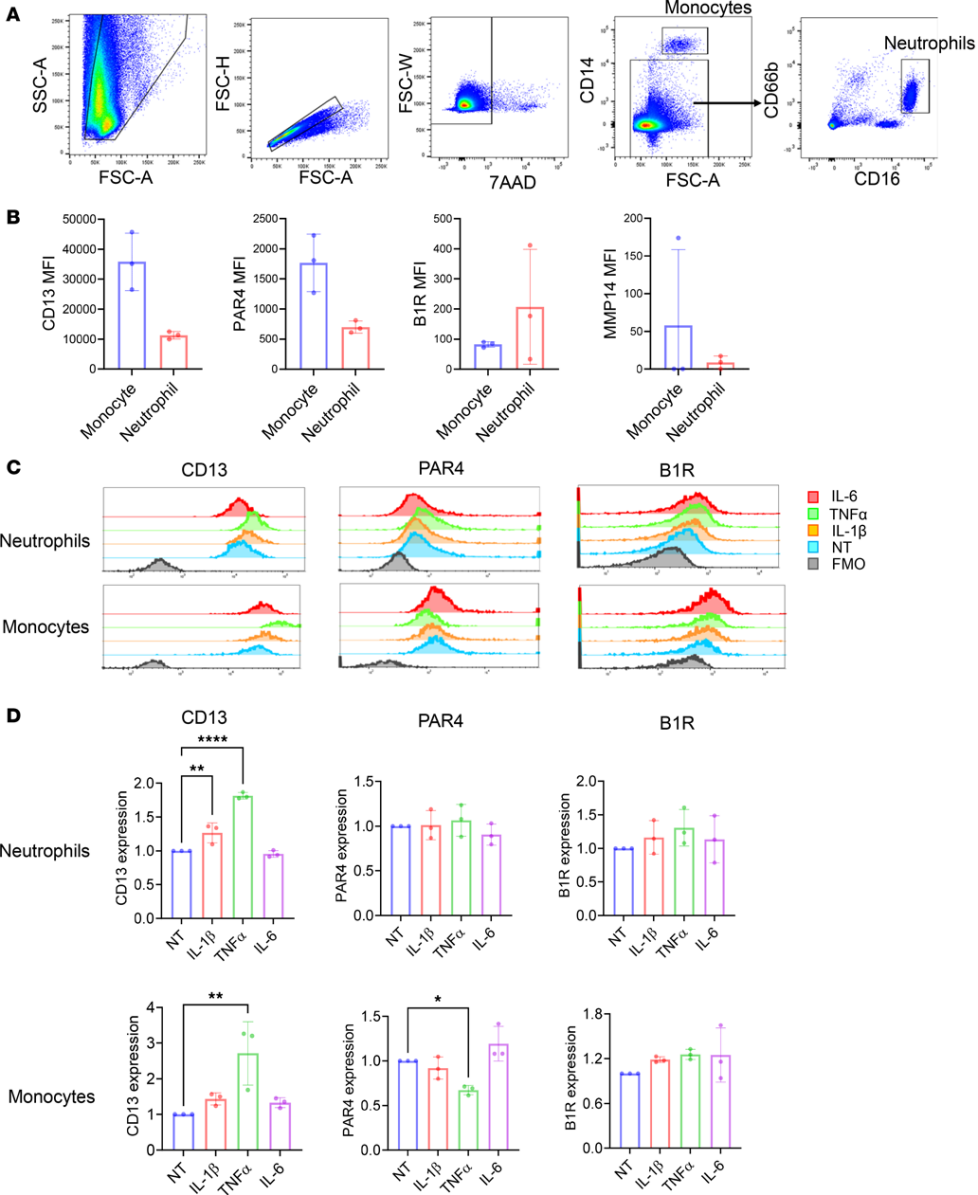
CD13, PAR4, and B1R are highly expressed on neutrophils and monocytes from healthy donors. (**A**) Gating scheme of flow cytometry analysis on monocytes and neutrophils. (**B**) CD13, B1R, and PAR4 were expressed on monocytes and neutrophils, while MMP14 showed minimal expression on these cells (*n* = 3). (**C**) Histograms showing the changes in CD13, PAR4, and B1R expression in neutrophils and monocytes with or without IL-1β, TNF-α, or IL-6 stimulation. (**D**) Quantification of relative expression of CD13, PAR4, and B1R on neutrophils and monocytes after stimulation with IL-1β, TNF-α, or IL-6 compared with unstimulated control from 3 healthy donors. Results are expressed as mean ± SD. *P < 0.05, **P < 0.01, ****P < 0.0001. Significance was determined by 1-way ANOVA. FMO, fluorescence minus 1 (gating control); NT, not treated.

To validate the results of the single-cell RNA-Seq analysis, we measured the expression of CD13, PAR4, B1R, and MMP14 on neutrophils and monocytes from healthy donors. The gating scheme for flow cytometry is shown in Figure 5A. All proteins were expressed on both cell types except for MMP14 (Figure 5B). To further determine the effect of pro-inflammatory cytokines on the expressions of CD13, PAR4, and B1R, we stimulated neutrophils and monocytes with IL-6, IL-1β, and TNF-α. In neutrophils, IL-1β and TNF-α significantly induced CD13 expression (Figure 5, C and D) while IL-6 had no effect. These cytokines had minimal effects on PAR4 and B1R expression on these cells. For monocytes, all cytokines were able to induce CD13 expression. TNF-α significantly downregulated PAR4 in these cells. In contrast, B1R expression on monocytes was induced by IL-1β and TNF-α. These results suggest the receptors for sCD13 are indeed present on neutrophils and monocytes.

**Figure 6:**
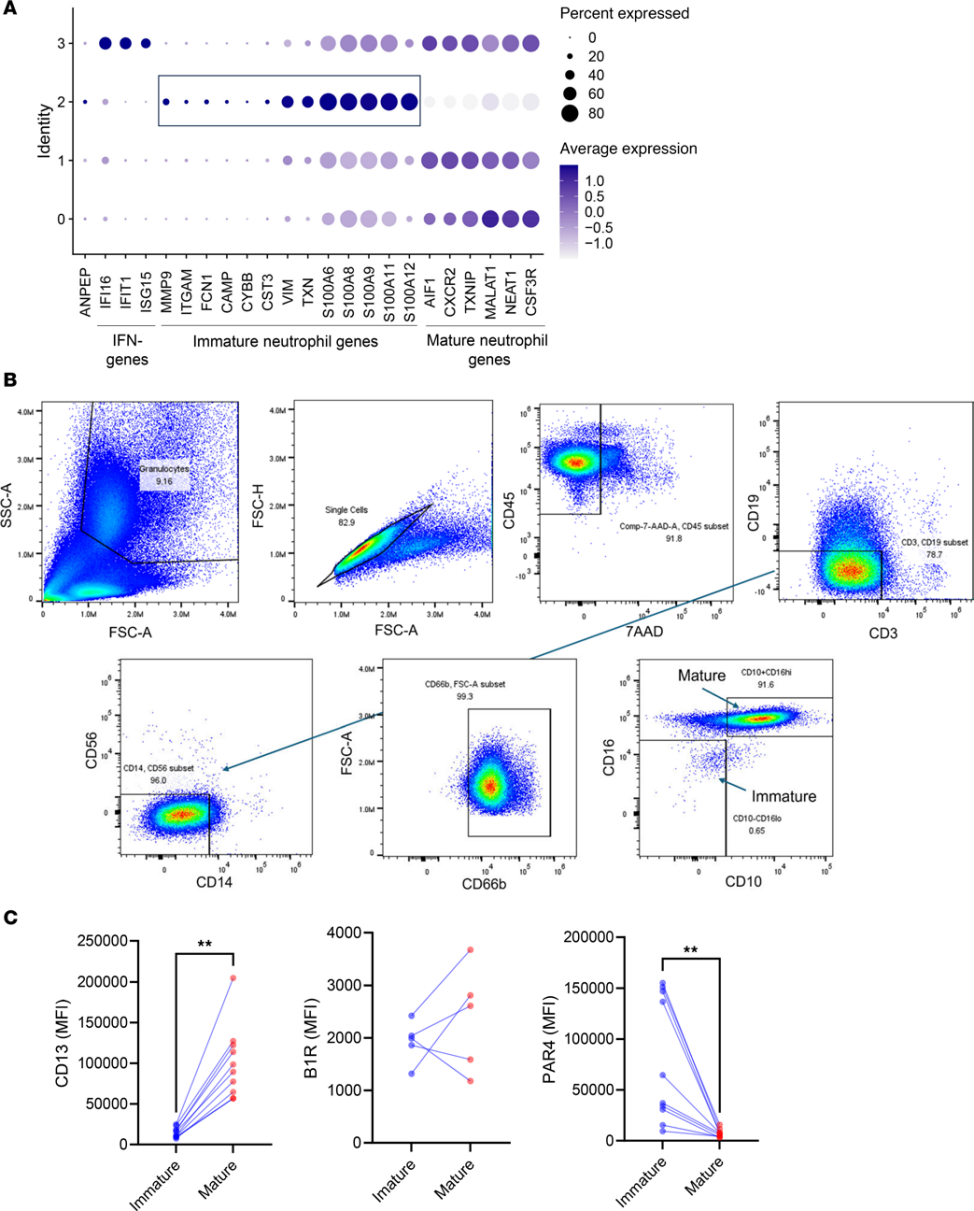
Divergent CD13 expression is observed in mature and immature neutrophils. (**A**) Reanalysis of single-cell RNA-Seq results generated from circulating human neutrophils (32) revealed that immature neutrophils have higher *ANPEP* expression. Dot plot of *ANPEP*, IFN, and neutrophil maturity genes for each neutrophil cluster, showing the average expression level and the percentage of cells expressing the gene in each cluster. (**B**) Gating scheme of flow cytometry analysis on mature and immature neutrophils isolated from whole blood. CD10^+^CD16^hi^ defines mature neutrophils while CD10^–^CD16^lo^ defines immature neutrophils. (**C**) Significantly higher expression of CD13 and lower expression of PAR4 were observed in mature neutrophils compared with immature neutrophils while B1R expression showed similar levels. Data generated from 5–10 healthy controls. Results are expressed as mean ± SD. **P < 0.01. Significance was determined by Wilcoxon’s test.

To further investigate the expression of CD13 and neutrophil maturity, we conducted an additional analysis utilizing a single-cell RNA-Seq dataset of human circulating neutrophils. This dataset was generated using a modified technical and analysis pipeline specifically designed for single-cell RNA-Seq from neutrophils (32). Consistent with previous findings, we identified 4 neutrophil clusters (Supplemental Figure 12). Among these, cluster 2 showed higher expression of genes associated with immature neutrophils (Figure 6A), while the other 3 clusters exhibited abundant expression of genes related to mature neutrophils. Notably, *MME*, which encodes the membrane metallo-endopeptidase CD10 — a marker for mature neutrophils — was more highly expressed in cluster 2 (Supplemental Figure 12B). Aligning with the published report (32), cluster 3 neutrophils exhibited significantly higher expression levels of type I IFN genes. In our analysis, *ANPEP* expression was most abundant in cluster 2, the cluster characterized by the highest expression of genes linked to neutrophil immaturity (Figure 6A), aligning with our analysis of COVID-19 datasets.

To determine if the effects of sCD13 differ between mature and immature neutrophils, we first examined the single-cell RNA-Seq data from Supplemental Figure 12 for B1R and PAR4 expression in the neutrophil clusters. Unfortunately, the expression levels of both receptors were too low to draw meaningful conclusions from the neutrophil transcriptome. Therefore, we proceeded by analyzing circulating neutrophils from healthy donors using flow cytometry. We used CD16 and CD10 markers to distinguish between mature and immature neutrophils (Figure 6B). Our analysis revealed that CD10^–^CD16^lo^ immature neutrophils constituted 0.99% ± 1.46% (range: 0.01%–4.82%) of the total neutrophil population in healthy controls. CD13 expression was consistently lower in immature neutrophils compared with mature neutrophils from the same donor (Figure 6C). In contrast, PAR4 levels were significantly lower in mature neutrophils while B1R did not differ significantly between the 2 populations. Together, these results suggest a discordance between mRNA and protein levels of CD13 in neutrophils. In addition, they indicate that the 2 receptors, PAR4 and B1R, may play different roles in sCD13’s effects on the 2 populations of neutrophils.

## Discussion

This study investigates the pivotal role of sCD13 in the inflammatory and immune responses associated with COVID-19, with particular attention paid to its impact on neutrophil function and disease severity. Elevated levels of sCD13 were found in the plasma of patients with COVID-19, correlating strongly with inflammation biomarkers, which suggests a substantial role for sCD13 in the hyperinflammatory states observed in severe cases of the infection. These findings align with the role of sCD13 in other diseases, including RA and systemic sclerosis (19, 20).

From this study, our data point to roles of sCD13 in COVID-19 through 2 potential mechanisms. One is promotion of inflammation, which culminates in some patients in cytokine storm. The second is activation of neutrophil migration, function, and NETosis. In COVID-19, the disproportionate release of cytokines, known as a cytokine storm, accounts for much of the severe inflammatory damage observed. sCD13 is implicated in this process by amplification of cytokine levels, especially IL-6, known to be induced by sCD13 (17, 20), which is crucial in driving this overactive immune response. In addition, sCD13 might synergize with other pro-inflammatory mediators and complement pathways, thereby indirectly promoting NETosis. Elevated levels of pro-inflammatory proteins such as TNF-α and IL-1β can induce the expression of PAR4 and B1R on immune and stromal cells, further amplifying the effect of sCD13.

Altered neutrophil phenotype, abundance, and functionality are found in patients with COVID-19. Elevated numbers of neutrophils have been reported in blood, nasopharyngeal epithelium, as well as distal parts of the lung (29, 33, 34). Through single-cell RNA-Seq, diverse neutrophil clusters with distinct transcriptional signatures have been identified, underscoring the emergence of immature and dysfunctional mature neutrophils, particularly in severe cases (35, 36). This shift suggests emergency granulopoiesis, a rapid production of neutrophil precursors, as a response to heightened demand during severe infection. Soluble mediators that affect neutrophil functions are also reported to be dysregulated in patients with SARS-CoV-2 infection. Neutrophil chemoattractants along with infiltrating neutrophils were found in infected lungs (37), and neutrophil mediators, including S100A8/A9 (calprotectin), are elevated in blood from patients with severe disease (36, 38). Moreover, NETs are elevated in plasma from patients with COVID-19 and correlate with disease severity and thrombotic events (25, 26). Autopsies also revealed NET enrichment in COVID-19 patient lungs (39). Pro-inflammatory mediators in the circulation, including autoantibodies, can enhance NETosis (40). This is supported by studies showing that serum from patients with COVID-19 induced NETosis (14, 25). These observations collectively provide the evidence that neutrophils play significant roles in COVID-19 progression and death.

sCD13 may play an influential role in these aforementioned neutrophil dynamics by inducing NETosis. Our in vitro findings reveal sCD13’s capability to stimulate NETosis, suggesting that elevated levels of sCD13 in patients with COVID-19 might contribute to the excessive NET formation linked to disease severity. This process could be mediated through sCD13’s interaction with its receptors, B1R and PAR4, which are more prevalently expressed on immature neutrophils. Given that endothelial activation and injury driven by NETs have been suggested as key pathogenic steps in COVID-19, we speculate that these processes could contribute to COVID-19 pathology through sCD13-induced NETosis. Moreover, we showed that sCD13 induces neutrophil chemotaxis, and together with NETosis, these are critical ways in which neutrophils respond to inflammatory cues. In lung tissues and peripheral immune cells from patients with COVID-19, cellular CD13 expression is both abundant and trends with disease severity. All these data point to the possibility that sCD13 plays a pathogenic role by attracting inflammatory cells and triggering NETosis. In addition, sCD13 might contribute to the accumulation of NETs and infiltrating neutrophils in the lungs. Given that MMP14 and CD13 are coexpressed on lung epithelial cells and macrophages (Figure 4 and Supplemental Figure 10), these cells could be the source of sCD13 in the lung, leading to significantly elevated local levels that attract and trap neutrophils and other immune cells in the tissue during infection. Indeed, our current and previous studies have shown that sCD13 promotes chemotaxis of monocytes and cytokine-activated T cells (17, 18).

In addition to modifying neutrophil accumulation, CD13 appears to associate with neutrophil maturity. Using a gene set associated with neutrophil immaturity (31), we identified immature neutrophils in the lungs and peripheral immune cells of patients with COVID-19, particularly those with severe disease, where these cells were more prevalent and showed high *ANPEP* expression. This was further validated with data from healthy donors (32), revealing that while immature neutrophils have increased *ANPEP* mRNA levels, their surface protein levels of CD13 are lower compared with mature neutrophils. Similarly, CD10, another maturation marker, shows elevated mRNA in immature neutrophils but lacks corresponding protein expression (32). The exact function of membrane-bound CD13 in neutrophil maturation is not fully understood. Since MMP14 expression is minimal in neutrophils, it is unlikely that they are the primary source of sCD13. sCD13 might also be exported via exosomes (16), a process observed in other cell types. Membrane-bound CD13 is linked to critical neutrophil functions such as phagocytosis, reactive oxygen species production, and NET formation (41), suggesting that its higher expression in mature neutrophils may enhance these activities. Furthermore, neutrophil elastase, a component of NETs, could modify CD13 by cleaving it at various sites, potentially affecting its aminopeptidase activity (42, 43). These interactions indicate a complex role for CD13 in modulating neutrophil function and suggest further investigation is necessary to understand its implications in COVID-19.

In addition to neutrophils, mast cells may also play a multifaceted role in COVID-19 (44). Their excessive activation can lead to a hyperinflammatory response, which is linked to severe COVID-19 symptoms and complications (45). Positioned predominantly in the respiratory tract and around blood vessels, mast cells are key players in the local inflammatory responses observed in the lungs of patients with COVID-19 (11). Notably, mast cell–specific proteases such as chymase and tryptase have been significantly correlated with disease severity and may contribute to PAR4 activation, exacerbating neutrophil activity. In addition, TNF-α and IL-6 produced by mast cells could enhance B1R expression on cells and promote sCD13 shedding in the lung.

The mechanisms by which sCD13 acts in COVID-19 involve receptor-mediated pathways and its tissue expression in the lung. sCD13 interacts with receptors B1R and PAR4 on immune cells including monocytes and neutrophils, suggesting a complex signaling network underpinning its pro-inflammatory effects. We showed that both B1R and PAR4 are expressed on monocytes and neutrophils, and their expression could be modified by pro-inflammatory cytokines. We further revealed that the effects of sCD13 on neutrophils appear to be mediated through these 2 receptors, as inhibitors of B1R and PAR4 blocked sCD13-induced NETosis and chemotaxis. Interestingly, we also observed that PAR4 expression on immature neutrophils is significantly higher compared with mature neutrophils, while B1R levels remain consistent across both populations. This suggests that sCD13 may exert stronger effects through PAR4 in immature neutrophils, particularly under the inflammatory conditions of COVID-19, where their numbers significantly increase (46). We also speculate that the effect of sCD13 through B1R on both mature and immature neutrophils in healthy controls would be similar and perhaps more pronounced under inflammatory conditions such as COVID-19, where it can be induced by cytokines. The lower PAR4 expression on mature neutrophils was unexpected, and it highlights a potential divergence in how sCD13 influences different neutrophil populations. Similar receptor expression patterns are observed in cytokine-activated T cells and ECs, which also express B1R and PAR4 (Supplemental Figures 10 and 13) (20), indicating that sCD13 affects multiple cell types. This receptor-mediated framework illustrates how sCD13 could modulate the behavior of immune and stromal cells, supporting the pro-inflammatory characteristic of COVID-19. Its broad influence across different cell types underscores the potential of targeting these pathways to mitigate the inflammatory responses associated with the disease.

The presence of CD13 in lung tissue highlights its local role in COVID-19 pathophysiology. Immunostaining of lung samples from patients with COVID-19 reveals high CD13 expression, which, along with the potential coexpression with MMP14, suggests that epithelial cells and macrophages could be primary sources of sCD13. In addition to significant increase of the sCD13 in patient blood, we showed that CD13 is expressed in COVID-19 patient lungs by immunostaining and by querying published datasets. Specifically, single-cell RNA-Seq analysis of lung samples and peripheral blood showed elevation of *ANPEP* expression at the transcription level in COVID-19 samples. Our analysis is further validated by published proteomic results. Multiorgan proteomic analysis of autopsy samples from COVID-19 patient organs revealed significant upregulation of CD13 in the lungs (47). In addition, sCD13 levels remained elevated significantly in bronchoalveolar lavage fluid from post–COVID-19 patients, defined as 3–6 months after hospital discharge with ongoing respiratory symptoms, accompanied by a significant increase in alveolar macrophages, T cells, B cells, and neutrophils (48). There was no significant difference of sCD13 in plasma comparing post–COVID-19 patients with healthy controls in this dataset. This is not surprising, as in a chronic autoimmune condition such as RA, sCD13 serum levels are not higher than those found in healthy controls, but synovial fluid sCD13 levels are significantly elevated compared with serum levels (18).

Sex, racial, and age differences significantly influence COVID-19 progression and outcomes. Males are known to experience higher mortality rates during acute COVID-19, while females are more susceptible to developing long COVID syndrome (49, 50). Immunologically, males exhibit elevated chemokines and cytokines, such as IL-8 and IL-18, along with higher monocyte counts, whereas females tend to exhibit a stronger T cell response (51, 52). This suggests that men generally mount a stronger innate immune response, whereas women display a more potent adaptive response to the virus. Genetic diversity also plays a critical role in COVID-19 susceptibility and severity, especially among different racial groups, including Black individuals (53, 54). Specific genetic variants, such as those in the HLA region and the angiotensin-converting enzyme 2 gene, can influence immune responses, inflammation, and coagulation. These genetic factors often interact with social determinants such as health care access, socioeconomic status, and living conditions, affecting the outcomes for these populations (55). In our study, it remains uncertain whether the observed differences in sCD13 levels among various subgroups are a cause or effect of COVID-19 severity. Age also influences sCD13 levels, which we found to be negatively correlated with age in both healthy controls and patients with COVID-19. Previous studies highlight that CD13 expression is higher in airway epithelial cells from older adults compared with younger individuals, potentially due to DNA methylation differences (56). This increase in CD13 may relate to higher SARS-CoV-2 recovery and varied IFN expression in older adults. In addition, the lower sCD13 levels in older people might be attributed to diminished cleavage activity by MMP14 (57, 58). However, the specific mechanisms through which age affects sCD13 levels warrant further exploration.

This study has several limitations that should be considered. The cross-sectional design of this study allows us to establish only associations but not causality. The plasma samples from the Michigan Medicine cohort consisted of remnant patient samples from the hospital, and therefore they were not frozen immediately after collection. However, we have established strong evidence of NETosis with these samples (25, 26) and acquired similar results from 2 other cohorts showing significant sCD13 elevation in patients with COVID-19. In addition, the ages of the healthy controls and the patients with COVID-19 were not perfectly matched. However, when segregated by age, we were able to show that sCD13 was significantly elevated in patients with COVID-19 compared with their corresponding controls in each age group (between 20 and 59 years old, Figure 1C). There are multiple pathways for platelet activation, many of which remained to be explored in the context of COVID-19 infection. Although we could not detect any role of sCD13 in direct activation of platelets, it is possible that it could indirectly participate in pathways through which other mediators activate platelet function. Along that line, although our study effectively outlines associations and potential mechanisms of action, conducting direct mechanistic experiments in vitro and using animal models would provide deeper insights into how sCD13 influences immune responses, neutrophil maturity, and thromboinflammation, thereby strengthening our findings.

In summary, these data suggest that CD13, both its membrane-bound and its soluble form, potentially assumes key roles in COVID-19, specifically in neutrophil function. Plasma sCD13 levels reflect the clinical condition of patients with COVID-19. The strong pro-inflammatory effects of sCD13 likely contribute to the unusual hyperinflammatory complications of this viral infection. Thus, sCD13 could indicate disease severity of COVID-19 while the receptors for sCD13 should be considered as potential therapeutic targets to treat complications of SARS-CoV-2 infection.

## Methods

### Sex as a biological variable.

This study included both men and women. Due to the differences identified in circulating sCD13 levels between the sexes, sex was treated as a biological variable.

### Patients and controls.

Serum samples were obtained from patients with a confirmed COVID-19 diagnosis hospitalized at Michigan Medicine (25). These samples were first used for clinical laboratory testing, with the remainder used for research. Some of the sera were stored at 4°C for up to 48 hours prior to storage at –80°C. Healthy controls were recruited through advertisement. Detailed participant characteristics are shown in Table 1. Two additional cohorts were also included for this study. Adult patients with COVID-19 described by Carmona-Rivera et al. (27) were included as the first validation cohort. The second validation cohort includes patients and unvaccinated healthy controls recruited from Weill Cornell Medical College (59, 60) (Supplemental Table 3). Plasma samples from this cohort were depleted of extracellular vesicles and particles by ultracentrifugation (61). The patients with sepsis were recruited from the University of Michigan (Supplemental Table 4).

### Quantification of sCD13 and cytokines.

sCD13 was quantified using the Human Aminopeptidase N/CD13 DuoSet ELISA from R&D Systems, Bio-Techne. Measurement of cytokines was done using the V-PLEX Human Cytokine 30-Plex Kit as described (27). The Cit-H3 levels in the patients with sepsis were measured by the human Cit H3 ELISA kit from Biorbyt.

### NETosis assay.

In all experiments, the sCD13 utilized was obtained from R&D Systems, Bio-Techne. This product was synthesized using a mouse myeloma cell line, NS0. The protein consists of the amino acid sequence spanning Lys69 to Lys967 of the full-length protein and has endotoxin levels below 1 EU/μg of protein. Neutrophils were isolated from healthy controls using heparinized tubes, followed by density-gradient centrifugation using Ficoll-Paque Plus (GE Healthcare, now Cytiva). Neutrophils were then purified using dextran sedimentation of the red blood cell layer, before lysing residual red blood cells with 0.2% sodium chloride. To assess NETosis, neutrophils were resuspended in RPMI medium (Gibco) and cultured in 96-well plates (1 × 10^5^/well) at 37°C with sCD13 (1 μg/mL, R&D Systems, Bio-Techne), thrombin (0.05 or 0.5 U/mL), or PMA (100 nM) in the presence or absence of B1R inhibitor SSR-240612 and PAR4 inhibitor BMS-986120. NETosis was assessed as previously described (62) by a SYTOX Green–based assay (Thermo Fisher Scientific), which detects extracellular DNA. After 3 hours of culture, SYTOX Green was added to a final concentration of 0.2 μM and incubated for an additional 10 minutes. Fluorescence was quantified at excitation and emission wavelengths of 485 nm and 520 nm, respectively, using a Cytation 5 Cell Imaging Multi-Mode Reader (BioTek, Agilent) with the following settings: light source: Xenon Flash; lamp energy: high; extended dynamic range read speed: normal; delay: 10 ms; measurements/data point: 10; read height: 7 mm.

In separate experiments, NETosis was assessed by measuring NET-associated MPO. After 3 hours of culture, the culture medium was discarded (to remove any soluble MPO) and replaced with 100 μL of RPMI supplemented with 10 U/mL Micrococcal nuclease (Thermo Fisher Scientific). After 10 minutes at 37°C, digestion of NETs was stopped with 10 mM EDTA. Next, supernatants were transferred to a V-shaped, 96-well plate and centrifuged at 350*g* for 5 minutes at room temperature to remove debris. Supernatants were then transferred into a new plate. An equal volume of 3,3′,5,5′-tetramethylbenzidine substrate (1 mg/mL, Thermo Fisher Scientific) was added to each well to measure MPO activity. After 10 minutes of incubation in the dark, the reaction was stopped by adding 50 μL of 1 mM sulfuric acid. Absorbance was measured at 450 nm using a Cytation 5 Cell Imaging Multi-Mode Reader.

### Immunofluorescence of NETs and neutrophils.

For immunofluorescence microscopy of NETs, neutrophils were seeded onto coverslips and stimulated with sCD13 (1 μg/mL, R&D Systems, Bio-Techne). Samples were then fixed with 4% paraformaldehyde, blocked, and incubated with primary antibody against neutrophil elastase (Abcam polyclonal ab21595). They were then incubated with FITC-conjugated secondary antibody (Southern Biotech 4050-02). DNA was stained with Hoechst 33342 (Thermo Fisher Scientific). Images were collected with a Cytation 5 Cell Imaging Multi-Mode Reader. In a separate experiment, neutrophils from healthy controls were isolated and seeded to Chamber slides (Thermo Fisher Scientific) and treated with sCD13 (1 μg/mL, R&D Systems, Bio-Techne). After an hour, cells were fixed and blocked, followed by incubation with either anti-PAR4 (F2RL3) (extracellular)-FITC antibody (Allomone Labs, polyclonal, APR-034-F) or anti-PAR4 antibody (clone 5F10, MilliporeSigma MABS1298). After incubating with secondary antibodies for the anti-PAR4 5F10 antibodies (A-11005, Invitrogen), the slides were mounted with ProLong Diamond Antifade mountant containing DAPI (Invitrogen).

### Platelet aggregation assay.

Platelets were isolated from healthy individuals as previously reported (63). Isolated platelets (2.5 × 10^8^) were stimulated with thrombin (0.025 U), PAR4 agonist peptide (Ala-Tyr-Pro-Gly-Lys-Phe-NH_2_ trifluoroacetate salt, 50 μM, MilliporeSigma), or sCD13 (1 μg/mL). Platelet aggregation was quantified using a Lumi-Aggregometer (Model 700D; Chrono-log), under stirring conditions (1,200 rpm) at 37°C for 10 minutes.

### Chemotaxis assay.

Neutrophil chemotaxis was performed using 48-well modified Boyden chambers. sCD13 (0.5–2 μg/mL) was added to the bottom wells of the chambers. IL-8 (10 ng/mL) and PBS solution served as positive and negative controls, respectively. Each test group was assayed in quadruplicate. Three high-power fields (×400) were counted in each replicate well. Chemotaxis assays using Incucyte (Sartorius) were performed following a previously described protocol (64). The top and bottom wells were coated with Matrigel (50 μg/mL) for 30 minutes at 37°C and 30 minutes at ambient temperature in advance. Neutrophils were pretreated with SSR-240612 (1 μM, Cayman Chemical) or BMS-986120 (10 μM, Cayman Chemical), respectively, for 60 minutes. Cells were then seeded in 60 μL RPMI medium with 1% FBS into the top wells and allowed to settle at 37°C for 60 minutes. The bottom wells were filled with 200 μL/well RPMI medium with sCD13 (1 μg/mL). IL-8 (10 ng/mL) or PBS solution served as positive and negative controls, respectively. Images were captured every hour for 6 hours, and migration was measured as the total remaining area covered by neutrophils on the top of the membrane.

### Flow cytometry.

Whole blood samples were collected from healthy donors, and red blood cells were removed by 3% dextran sedimentation for 30 minutes at room temperature. White blood cells in the upper phase were washed with PBS containing 2 mM EDTA and 2% FBS and stained with fluorochrome-conjugated antibodies against human CD3 (PE-Cy7, BioLegend 300420), CD13 (PE, BioLegend 301703), CD14 (APC-Cy7, BioLegend 301820) CD16 (PerCPCy5.5, Tonbo 615-1619-T025), CD19 (BV510, BioLegend 302241), CD45 (APC-Cy7, Tonbo 25-0459-T100), CD56 (PE, BioLegend 362508), CD10 (Super Bright 702, Invitrogen 67-010-642), PAR4 (FITC, Alomone Labs APR-034-F), B1R (APC, LS Bio LS-C275585), MMP14 (APC, R&D Systems, Bio-Techne; FAB9181A), and CD66b (Pacific Blue, BioLegend 305111). The stained cells were then analyzed using BD FACSCanto II Flow Cytometer or Cytek Aurora Spectral Analyzer followed by FlowJo software. Live neutrophils were defined as 7AAD^–^CD3^–^CD19^–^CD56^–^CD45^lo^CD16^+^CD66b^+^, while live monocytes were defined as 7AAD^–^CD3^–^CD19^–^CD56^–^CD45^hi^CD14^+^. Mature and immature neutrophils were further defined using CD10 and CD16. For cell treatment, whole blood samples from healthy donors were diluted 1:1 with RPMI and incubated with or without 10 ng/mL of IL-1β, TNF-α, or IL-6 for 18 hours. The resulting cells were collected as follows: nonadherent cells were first collected, and the attached cells were then collected by incubating with PBS containing 2 mM EDTA and 2% FBS at 37°C for 10 minutes before washing out the cells, followed by combining with the suspending cells. Red blood cells were removed before the resulting white blood cells were used for antibody staining and flow cytometry.

### Tissue staining.

Lung tissues were obtained from autopsies performed on patients with cause of death attributed to COVID-19 (39). Lung samples were fixed in formalin, embedded in paraffin wax, sectioned at 4 μm, placed onto charged microscope slides, and dried for 45 minutes at 60°C in a tissue drying oven. Dried slides were deparaffinized in xylene and rehydrated with sequential washes of alcohol and distilled water. These specimens were incubated with the following antibodies: Histone H3 (citrulline R2 + R8 + R17, rabbit polyclonal; Abcam ab5103), neutrophil elastase (rabbit polyclonal; MilliporeSigma 481001), and CD13 (clone 2F10, LSBio, LS-B14324, or clone EPR4058, Abcam ab108310) followed by incubation with secondary antibody using an Opal Multiplex IHC Kit (PerkinElmer). The samples were mounted with ProLong Diamond Antifade mountant containing DAPI. In a separate experiment, hematoxylin and eosin staining of the slides was conducted.

### Single-cell RNA-Seq analysis.

Data were extracted from single-cell RNA-Seq studies generated from lungs (National Center for Biotechnology Information [NCBI] Gene Expression Omnibus [GEO] GSE171524), upper airways (https://doi.org/10.6084/m9.figshare.12436517), immune cells (NCBI GEO GSE174072) from blood in healthy individuals or patients with COVID-19, or circulating neutrophils from healthy individuals (28–30, 32). Briefly, the datasets were read and further integrated using Seurat v4.0 (https://satijalab.org/seurat/index.html) (65). The expression data were scaled and visualized with uniform manifold approximation and projection. FeaturePlot function was used to show the expression of interested genes among cells. Average expression levels of *ANPEP* (gene encoding CD13) of patients with COVID-19 were calculated, and the median was used to divide the individuals into 2 groups: *ANPEP*-high and *ANPEP*-low. Differentially expressed genes were calculated with the function FindMarkers, then visualized with the EnhancedVolcano function. Pathway enrichment was performed using Metascape (https://metascape.org/gp/index.html#/main/step1) (66). Neutrophil-immaturity score was obtained using the AddModuleScore function with genes related with neutrophil immaturity (Supplemental Table 5) and shown with the VlnPlot function. Briefly, the AddModuleScore function could calculate average expression levels of featured genes on single-cell level, subtracted by the aggregated expression of control feature sets. All analyzed features are binned based on averaged expression, and the control features are randomly selected from each bin. The neutrophil-immaturity gene signature was obtained from previous studies comparing gene expression of immature neutrophils with mature ones (31, 67) and summarized in Supplemental Table 5. The heatmap was visualized using the web tool ClusterVis (https://biit.cs.ut.ee/clustvis/) (68).

### Statistics.

A normality test was first performed to determine the distribution of the data. To determine the differences in groups, Student’s 2-tailed *t* test, Mann-Whitney *U* test, Kruskal-Wallis test, Wilcoxon’s test, or 1-way ANOVA was performed using GraphPad Prism version 10. Correlation tests were carried out using the Correlation function in GraphPad Prism version 10, and the results were presented as Spearman’s correlation coefficient. *P* values of less than 0.05 were considered statistically significant. Results were expressed as mean ± SD.

### Study approval.

All individuals were enrolled in protocols approved by Institutional Review Boards from the University of Michigan and Weill Cornell Medicine. Written informed consent was obtained from participants before study participation.

### Data availability.

The values underlying graphed data and reported means in this study are available in the associated Supporting Data Values file. Sequencing data analyzed in this manuscript were obtained from published datasets deposited to the NCBI GEO, referenced in Methods, Results, and figures.

## Author contributions

All authors participated in the interpretation of study results and in the drafting, critical revision, and approval of the final version of the manuscript. PST, JSK, and DAF conceived the study. PST, RAA, GS, CL, CCR, SL, QW, PLC, MGR, KM, SEF, WDB, MNM, CF, AT, SY, YI, MAA, KK, BMF, EC, Y Zuo, LI, AC, FC, VQ, AS, DBK, DF, RC, OMD, HK, Y Zhang, MDM, HS, LN, RLZ, MM, YMD, IRM, MS, DL, YK, and MJK contributed to the acquisition of study results. PST, RAA, QW, KM, MGR, and CL performed the analysis of study results. PST, JSK, and DAF drafted and edited the manuscript.

## Supplementary Material

Supplemental data

Supporting data values

## Figures and Tables

**Table 1:**
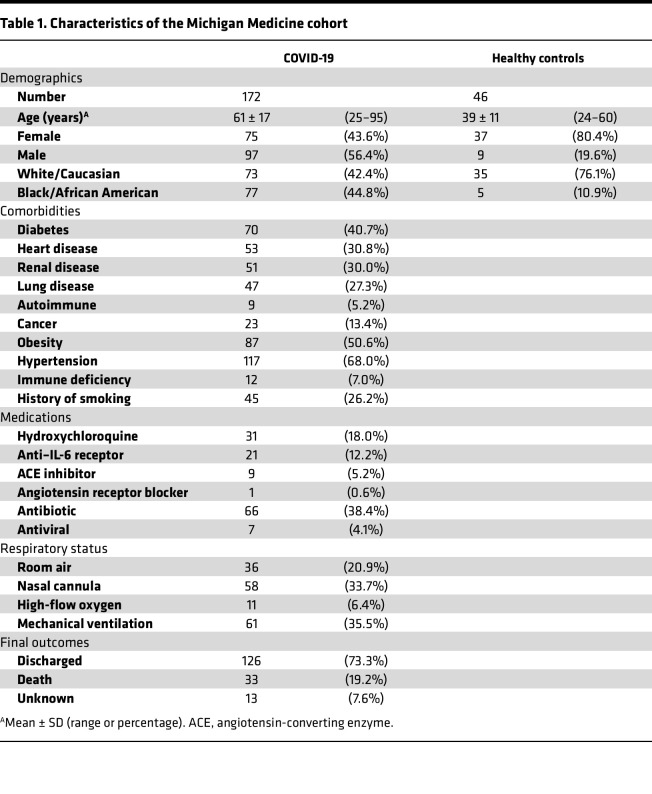
Characteristics of the Michigan Medicine cohort

## Version 1 04/01/2025

In-Press Preview

## Version 2 05/08/2025

Electronic publication
