# Supplementary material for: Soluble CD13 is a potential mediator of neutrophil-induced thrombogenic inflammation in SARS-CoV-2 infection
Source: JCI Insight. 2025 Apr 1;10(9):e184975. doi: 10.1172/jci.insight.184975 (PMC12128962; doi:10.1172/jci.insight.184975)
Supplement: Supplemental data [file jciinsight-10-184975-s026.pdf]

Supplemental Files for CD13-COVID-19 manuscript

Supplemental Figure 1. Comparison of male and female patients hospitalized for COVID-19. (**A**) sCD13 levels in male and female patients. The correlation was stronger in male patients with parameters of inflammation (**B**) and markers for NETosis (**C**). Results are expressed as mean +/- SD in (A) and p<0.05 was considered significant.

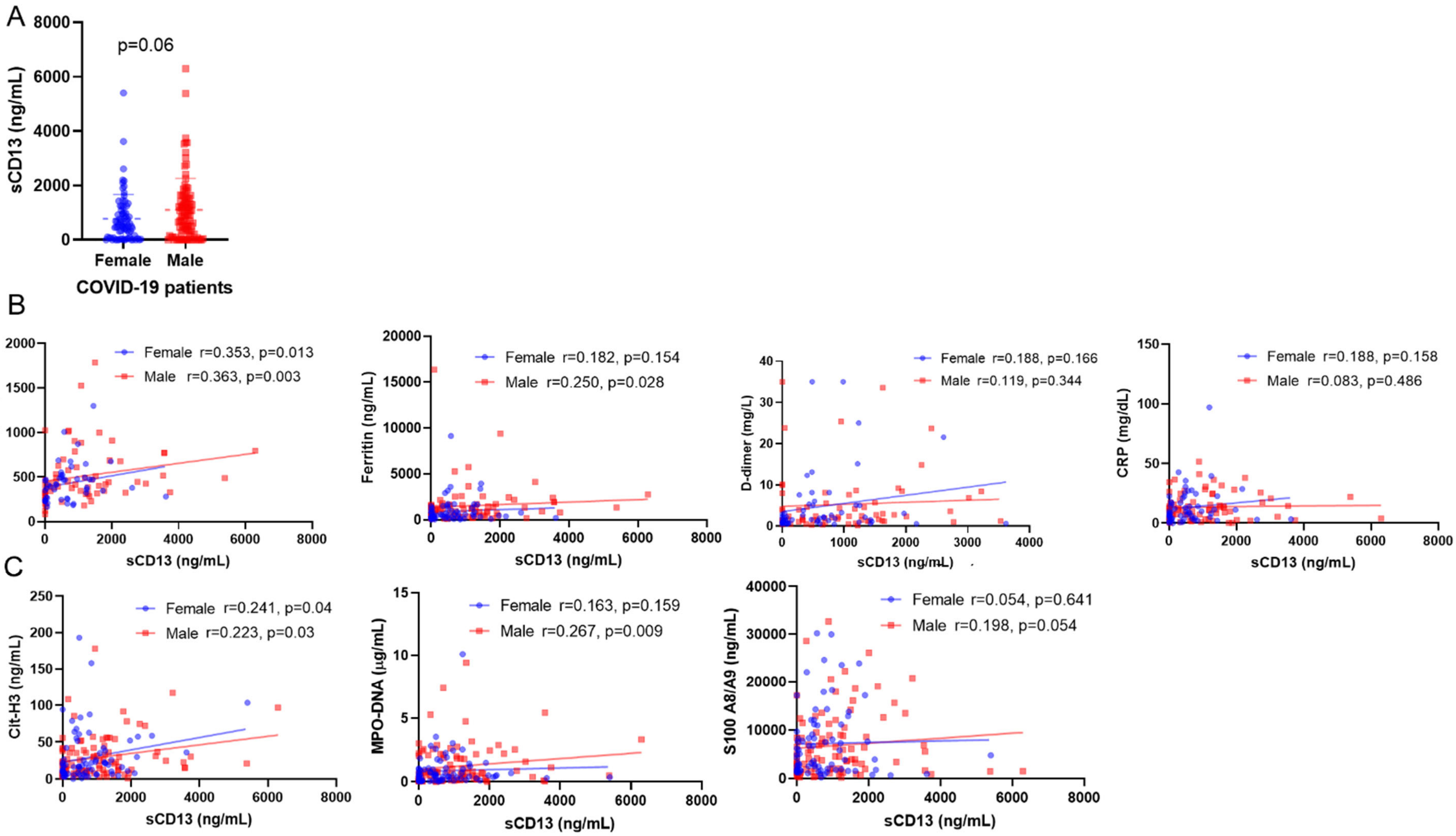

Supplemental Figure 2. A negative correlation between sCD13 and age was found in both healthy controls and COVID-19 patients.

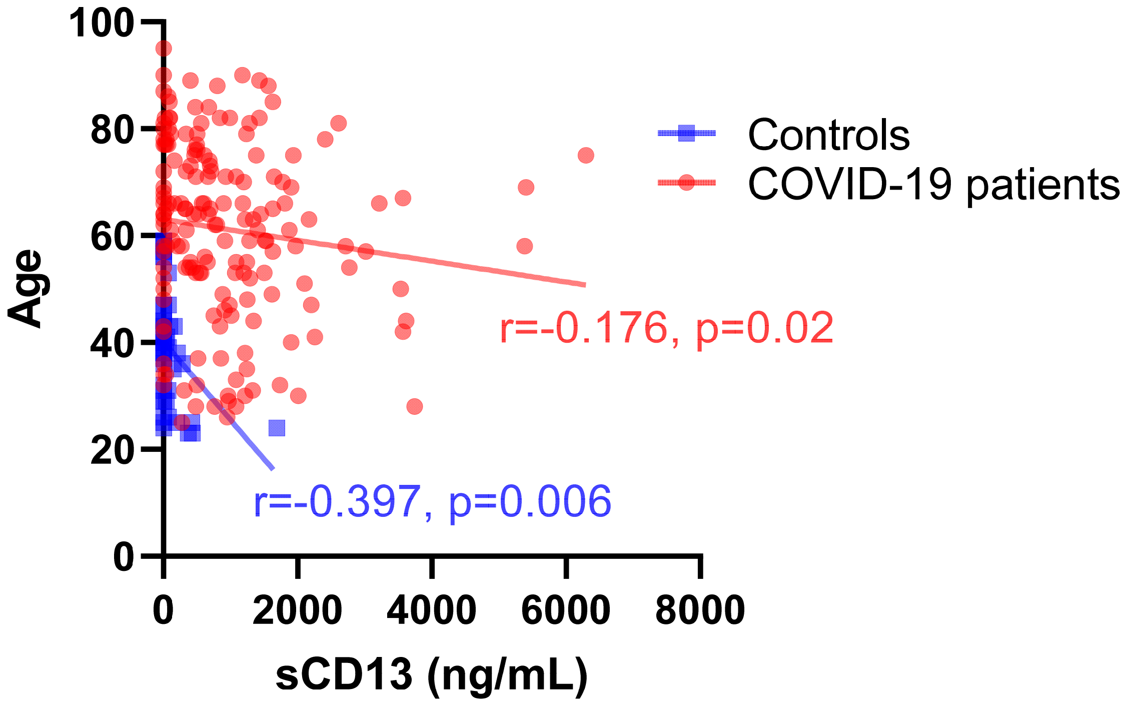

Supplemental Figure 3. The levels of serum sCD13 were similar among patients with different clinical outcomes, including death or discharged. Status “unknown” indicates absence of follow-up information. Results are expressed as mean +/- SD.

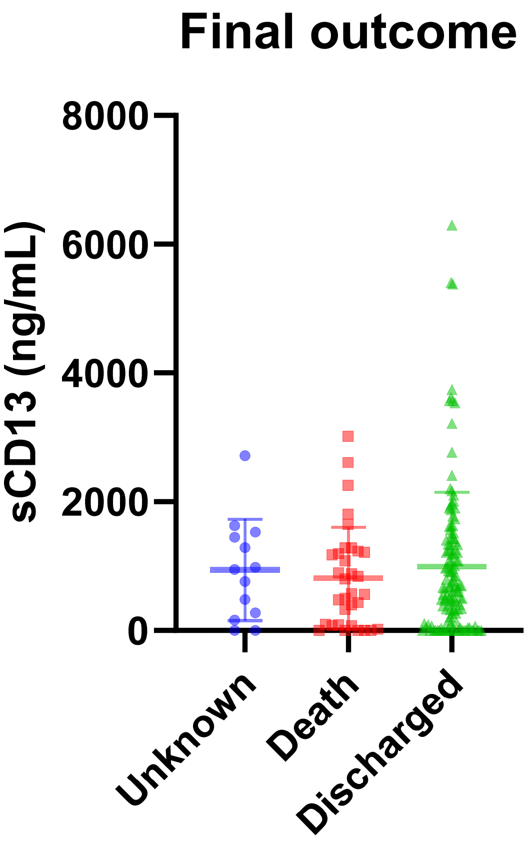

Supplemental Figure 4. Serum sCD13 levels in black patients with COVID-19 showed stronger correlation with inflammatory factors (**A**) compared to white patients, while levels from white patients showed stronger correlation with NETosis markers compared to black patients (**B**).

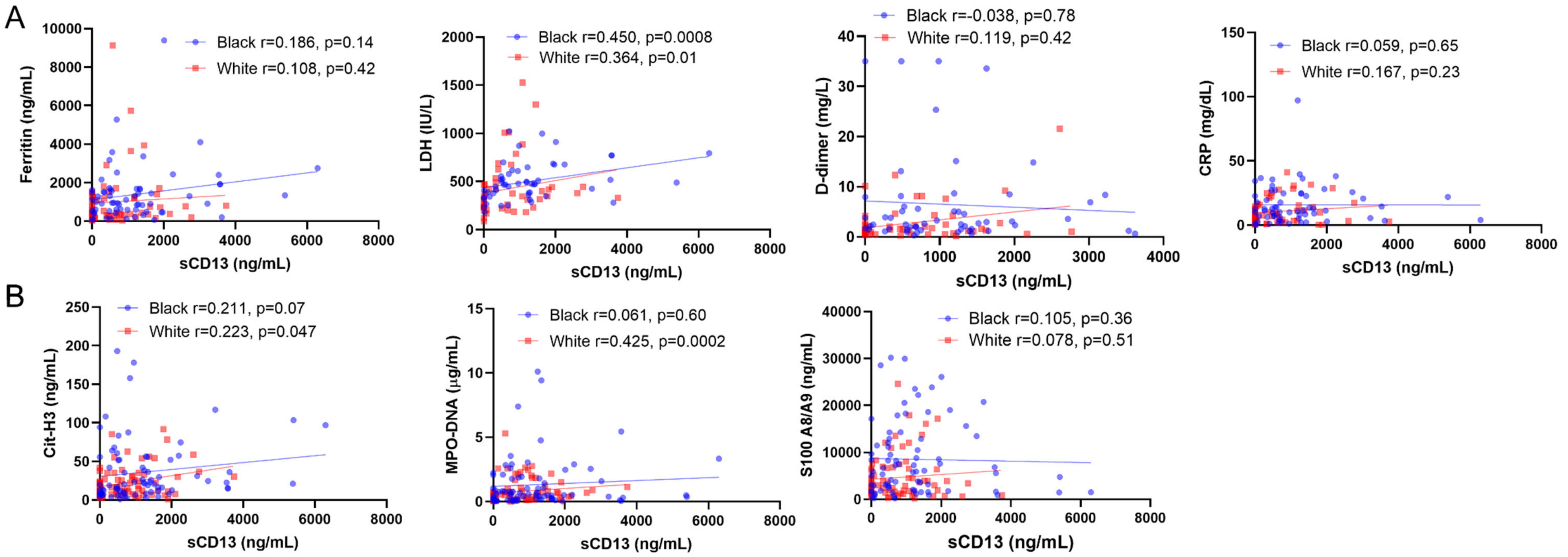

Supplemental Figure 5. Serum sCD13 levels in patients with COVID-19 (with Omicron variant) showed strong correlation with inflammatory cytokines.

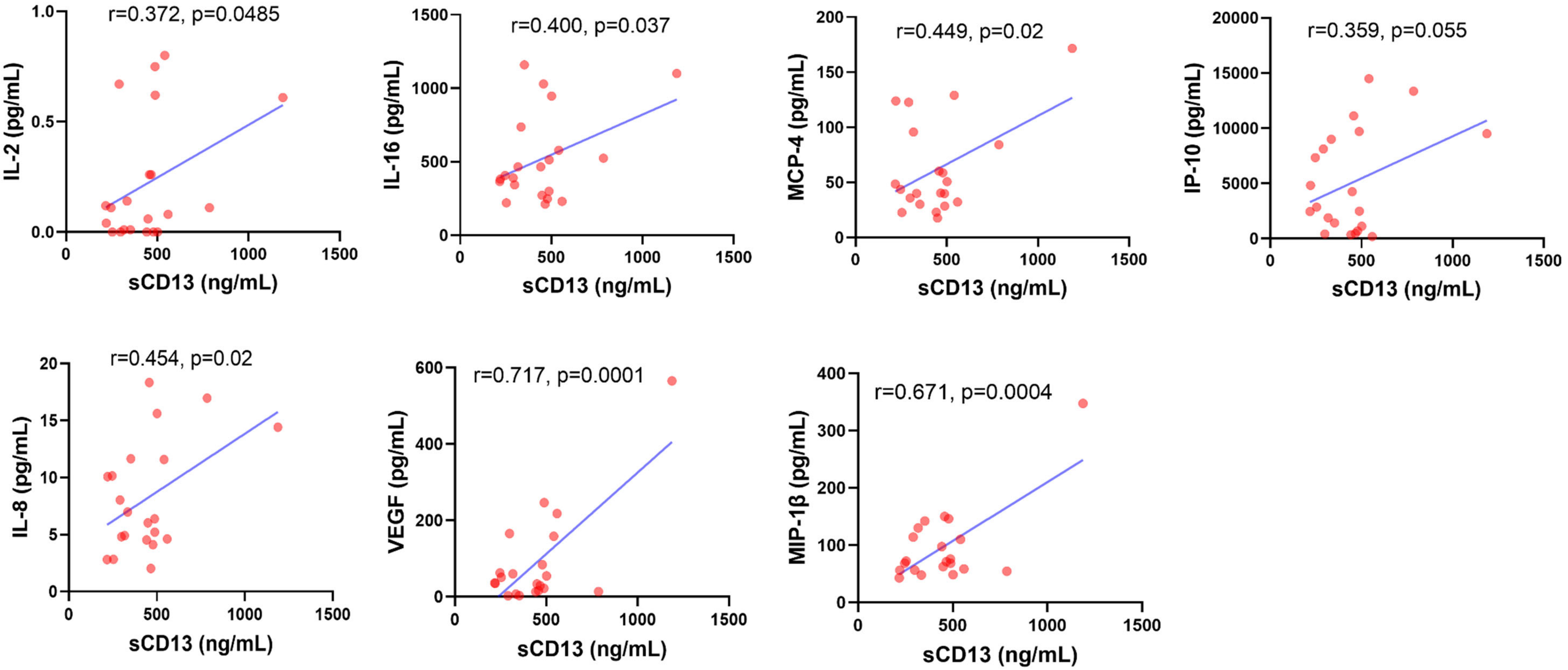

Supplemental Figure 6. Serum sCD13 levels in sepsis patients showed elevated levels of sCD13 compared to controls (**A**). sCD13 did not correlate with cit-H3 or S100A8/A9 levels in this cohort (**B**). Results are expressed as mean +/- SD.

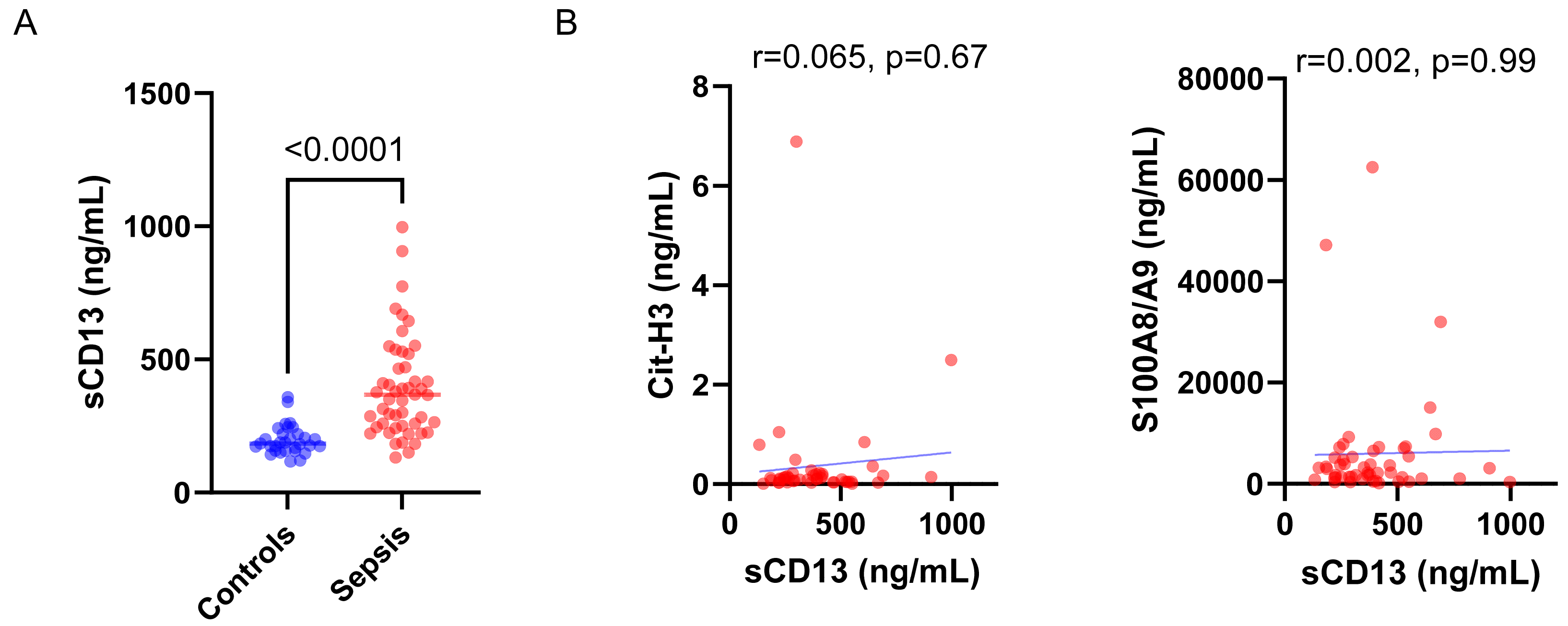

Supplemental Figure 7. sCD13 promotes neutrophil NETosis and chemotaxis but not platelet aggregation. sCD13 induced NETosis as quantified using the cell-impermeant dye SYTOX Green or nuclease-liberated MPO activity. Phorbol 12-myristate 13-acetate (PMA) was used as a positive control (**A**). Treating neutrophils with both inhibitors showed an additive effect on sCD13-induced NETosis (**B**). sCD13 dose-dependently induced neutrophil chemotaxis compared to PBS control using a modified Boyden chamber. IL-8 was used as a positive control (**C**). In a separate experiment, neutrophil chemotaxis was measured using IncuCyte®. sCD13 induced neutrophil chemotaxis was blocked consistently by PAR4 inhibitor BMS-986120 as compared to the B1R inhibitor SSR-240612 (**D**). sCD13 did not induce platelet aggregation while a PAR4 agonist peptide (Ala-Tyr-Pro-Gly-Lys-Phe-NH_2 trifluoroacetate salt) or thrombin did (**E**). Thrombin did not induce NETosis (**F**). Results are expressed as mean +/- SD and p<0.05 was considered significant.

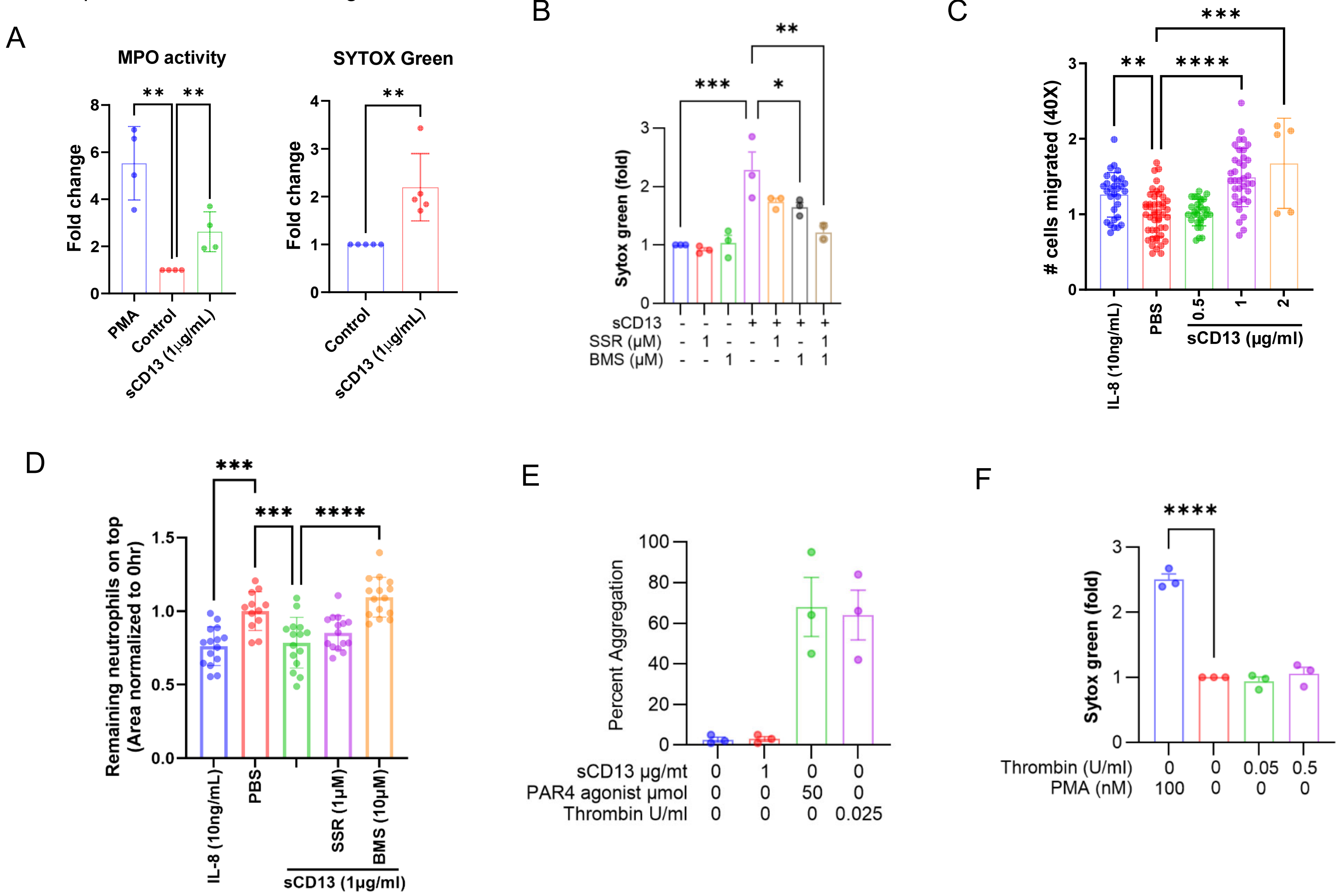

Supplemental Figure 8. Immunofluorescence and H&E staining was performed on lung tissues obtained from autopsies performed on patients with cause of death attributed to COVID-19, targeting CD13 (green), neutrophil elastase (red), and nuclear staining DAPI (blue).

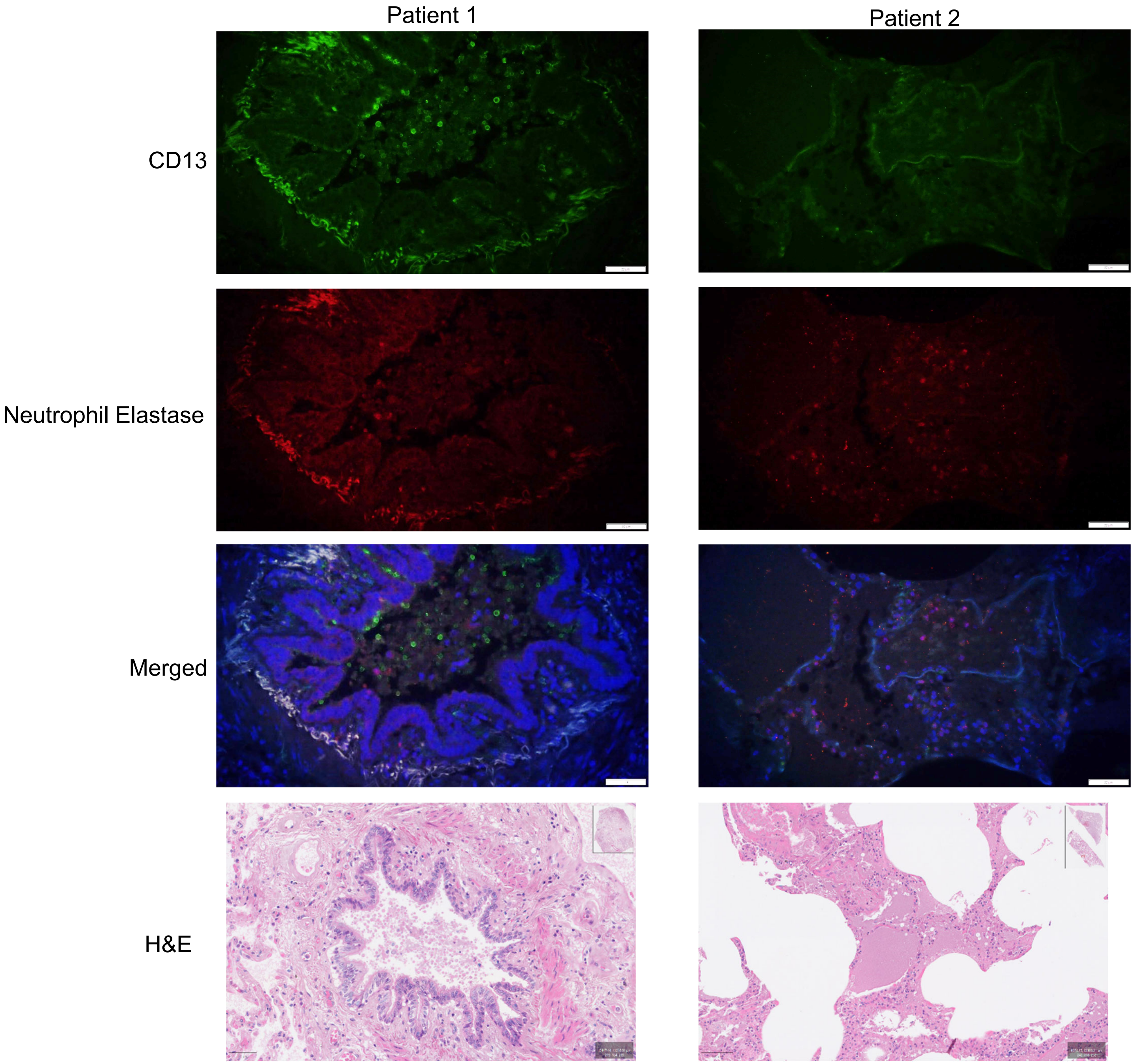

Supplemental Figure 9. Single-cell RNA-seq results of nasopharyngeal/pharyngeal swabs, bronchial brushings and bronchial lavages were generated from patients with COVID-19 (n=19) and healthy controls (n=5). Data is extracted from Chua et al. (**A**) UMAP displaying all identified cell types and disease state. (**B**) Both *ANPEP* and *MMP14* are expressed on various epithelial cells and macrophages. *ANPEP* is also expressed on neutrophils while *MMP14* is expressed on mast cells. *BDKRB1* (codes for B1R) is expressed on some epithelial cells with the highest expression on secretory cells and ciliated cells. *F2RL3* (codes for PAR4) was barely detected in this dataset.

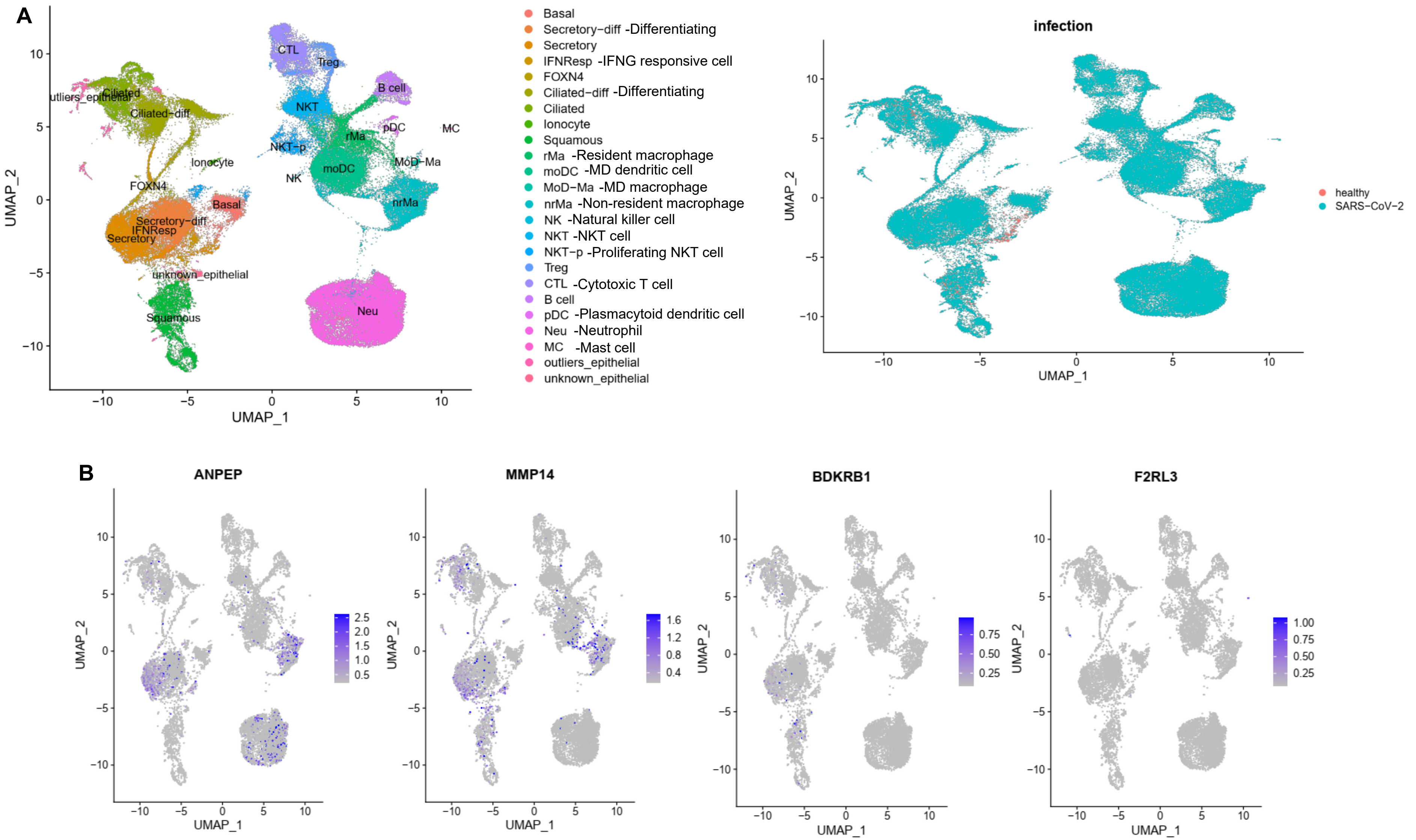

Chua, R.L., Lukassen, S., Trump, S. et al. COVID-19 severity correlates with airway epithelium–immune cell interactions identified by single-cell analysis. Nat Biotechnol 38, 970–979 (2020). https://doi.org/10.1038/s41587-020-0602-4

Supplemental Figure 10. Single-cell RNA-seq results of lung tissues were generated from patients who died with COVID-19 and underwent rapid autopsy (n=19) and non-COVID-19 controls (n=7). Violin plots of *ANPEP*, *MMP14*, *BDKRB1*, and *F2RL3* expression are shown in all identified cell types. Both *ANPEP* and *MMP14* are expressed on various epithelial cells, fibroblasts, NK/T cells, and macrophages/monocytes. *F2RL3* is predominantly expressed on endothelial cells, neuronal cells and smooth muscle cells. *BDKRB1* is expressed on some epithelial cells and fibroblasts. Data is extracted from Melms et al.

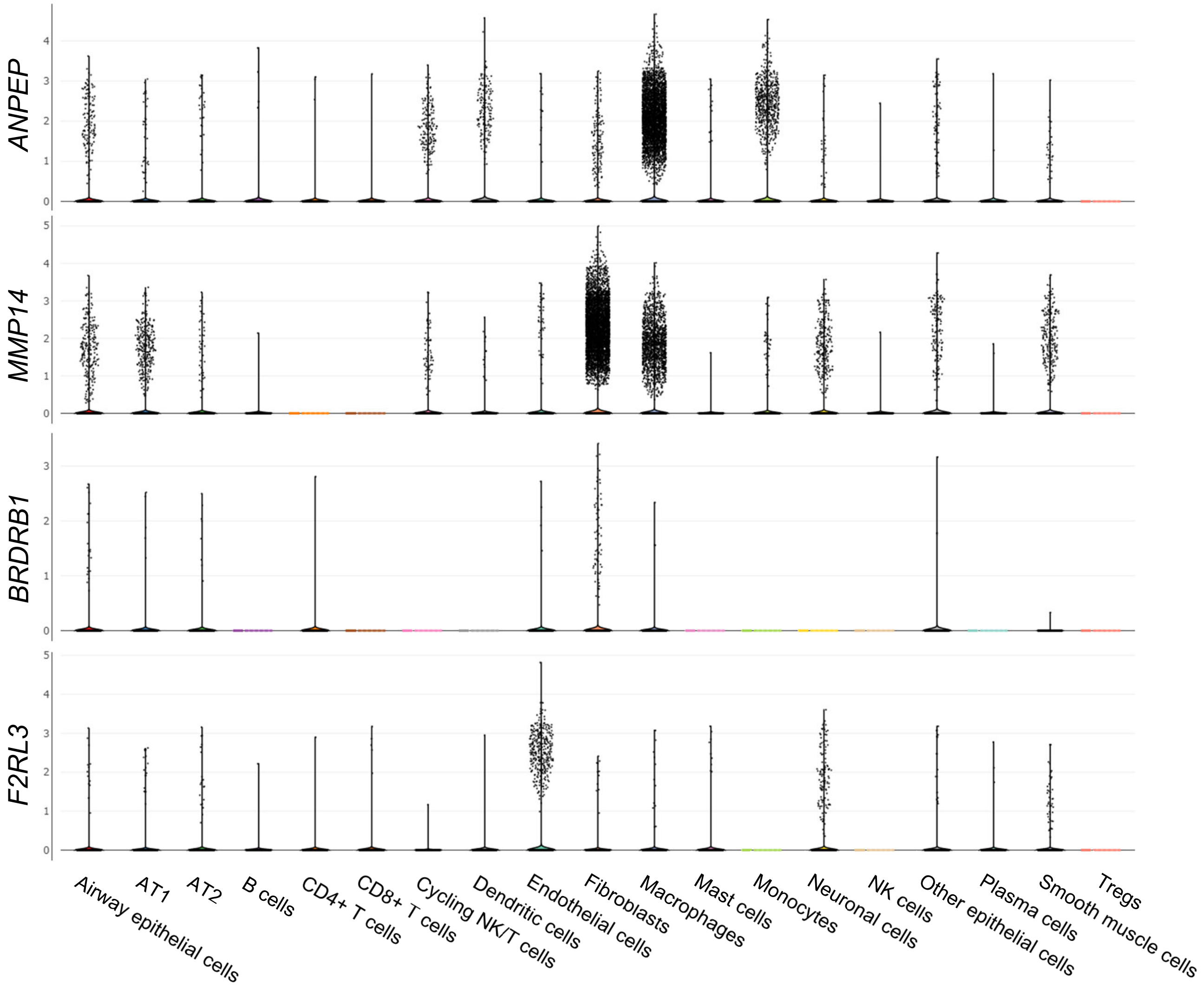

Melms, J.C., Biermann, J., Huang, H. et al. A molecular single-cell lung atlas of lethal COVID-19. Nature 595, 114–119 (2021). https://doi.org/10.1038/s41586-021-03569-1

Supplemental Figure 11. Peripheral immune cells from 33 COVID-19 patients and 8 healthy controls were subjected to single-cell RNA-sequencing. Data is extracted from Wilk et al. (**A**) Patients with higher *ANPEP* expression levels in immune cells appeared to have more severe disease based on the WHO severity score for COVID-19. (**B**) Using the median expression level of *ANPEP* in patients, the COVID-19 patients were divided into *ANPEP*-high and *ANPEP*-low populations. The volcano plot of the differentially expressed genes in neutrophils of the two groups are shown. (**C**) Pathway analysis of the differentially expressed genes in neutrophils of *ANPEP*-high and *ANPEP*-low patients. (**D**) The neutrophils from *ANPEP*-high COVID-19 patients show a gene signature of immature-like neutrophils characterized by the overexpression of genes encoding for several granule-containing proteins. HC: healthy controls

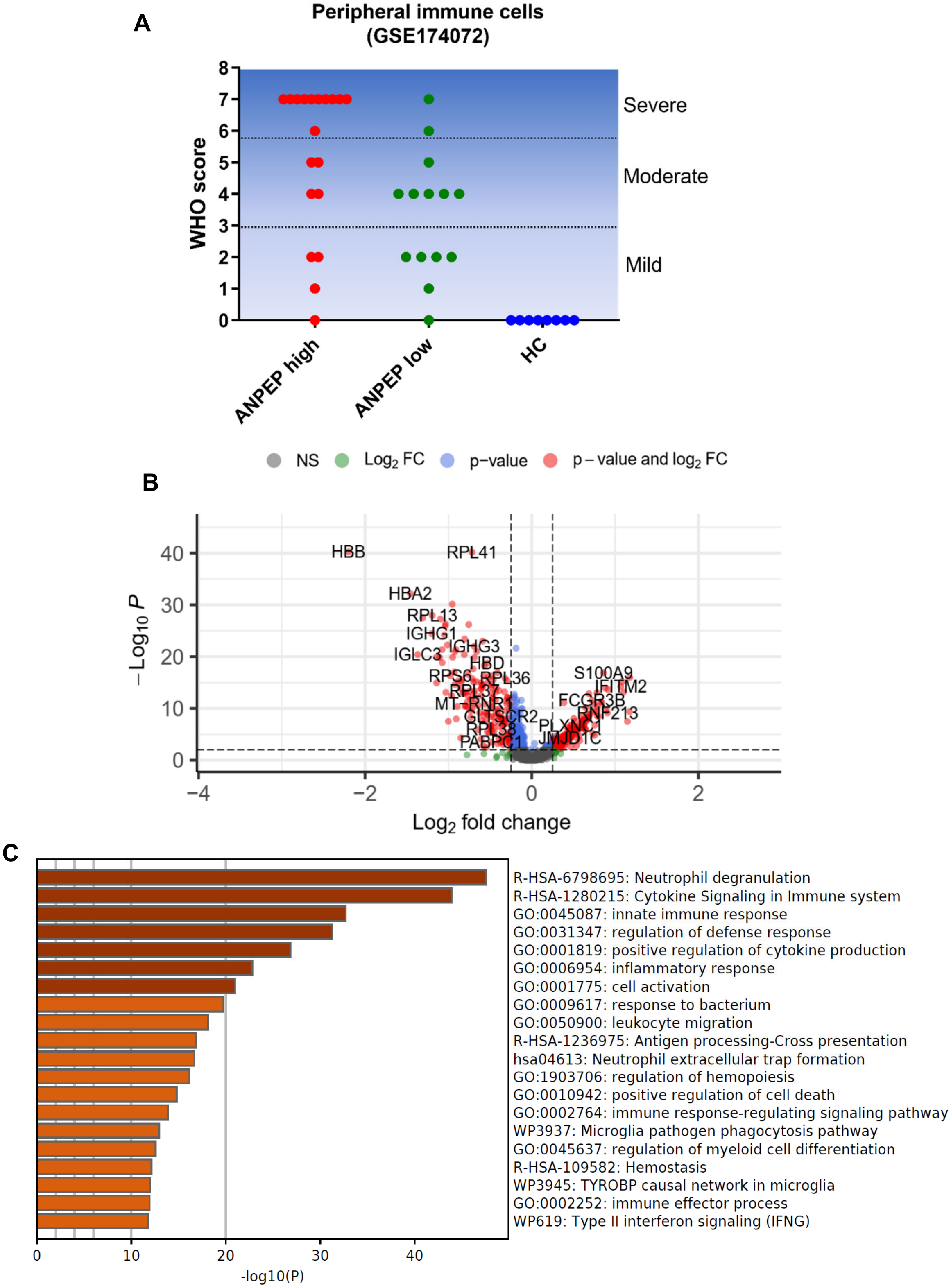

D

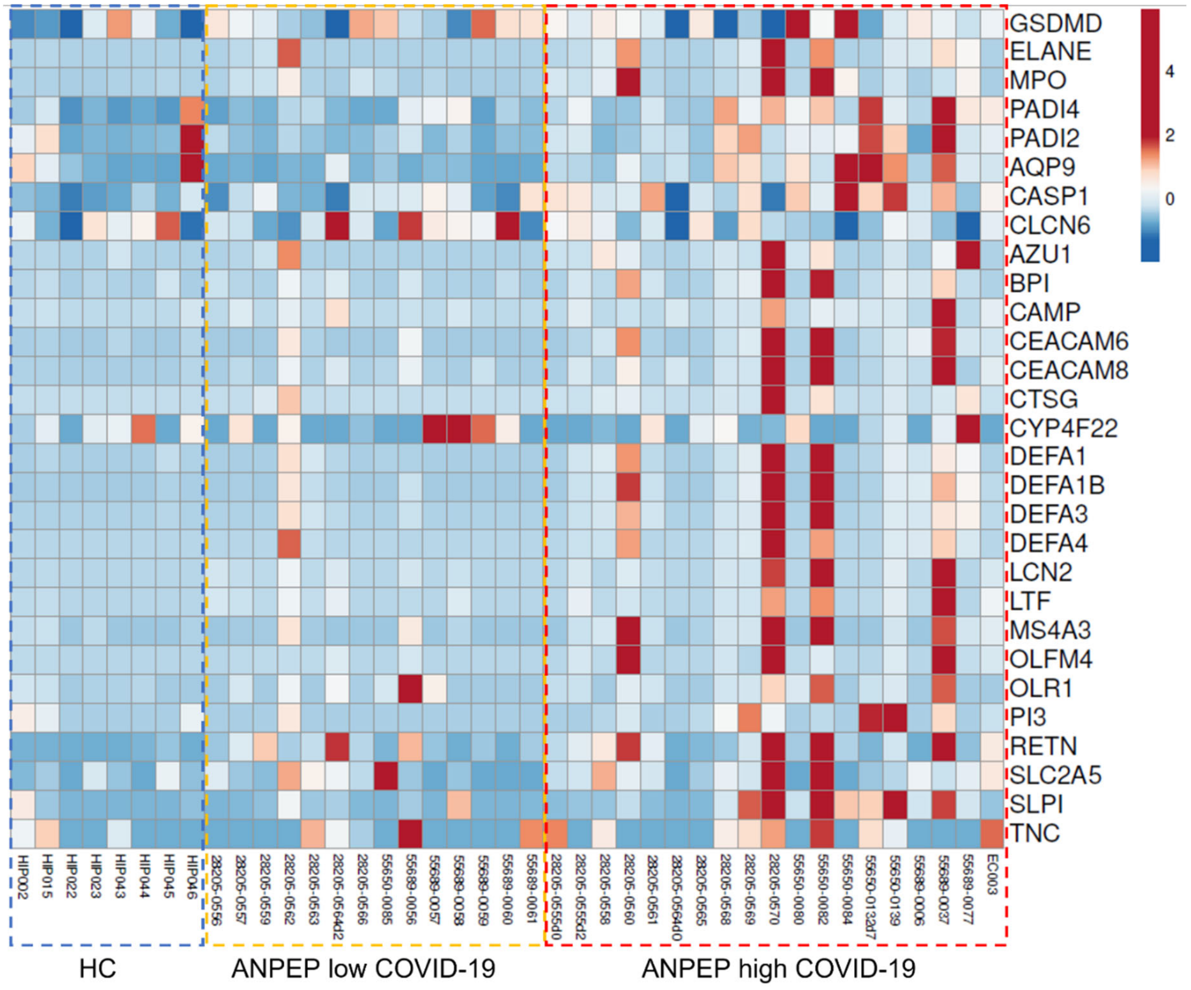

Wilk AJ, Lee MJ, Wei B et al. Multi-omic profiling reveals widespread dysregulation of innate immunity and hematopoiesis in COVID-19. J Exp Med 2 August 2021; 218 (8): e20210582. doi: https://doi.org/10.1084/jem.20210582

Supplemental Figure 12. Reanalysis of single-cell results generated from circulating human neutrophils revealed that immature neutrophils have higher *ANPEP* expression. Data is extracted from Wigeblad et al. (**A**) UMAP displaying all identified neutrophil clusters. (**B**) Heatmap of the top marker genes from each cluster. The cells corresponding to each cluster are grouped, as indicated by the colored bars.

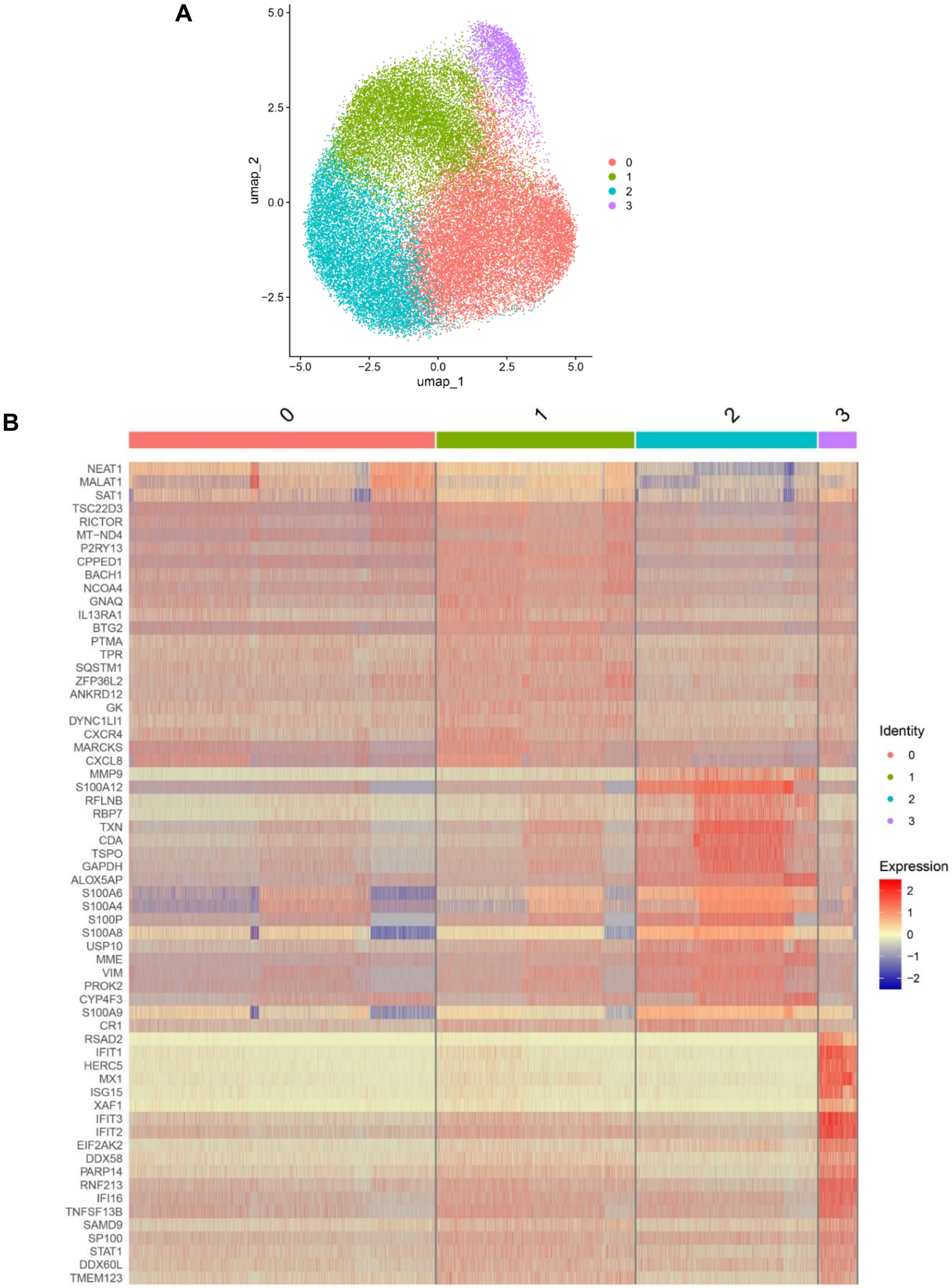

Wigerblad G, Cao Q, Brooks S et al. Single-cell analysis reveals the range of transcriptional states of circulating human neutrophils. J Immunol. 2022 Aug 15;209(4):772-782. doi: 10.4049/jimmunol.2200154

Supplemental Figure 13. B1R and PAR4 are expressed on cytokine-activated T cells (Tcks). T cells were purified from 3 healthy controls. Cytokine activated T cells were established by stimulating T cells with 25 ng/ml IL-2, 25 ng/ml TNFα, and 100 ng/ml IL-6 in RPMI1640 medium containing 10% fetal bovine serum, 2 mM L-glutamine for 8 days. B1R, and PAR4 expression on $CD4^+$ and $CD8^+$ T cells were done by flow cytometry. Results are expressed as mean +/- SD and $p<0.05$ was considered significant. N=3 healthy donors.

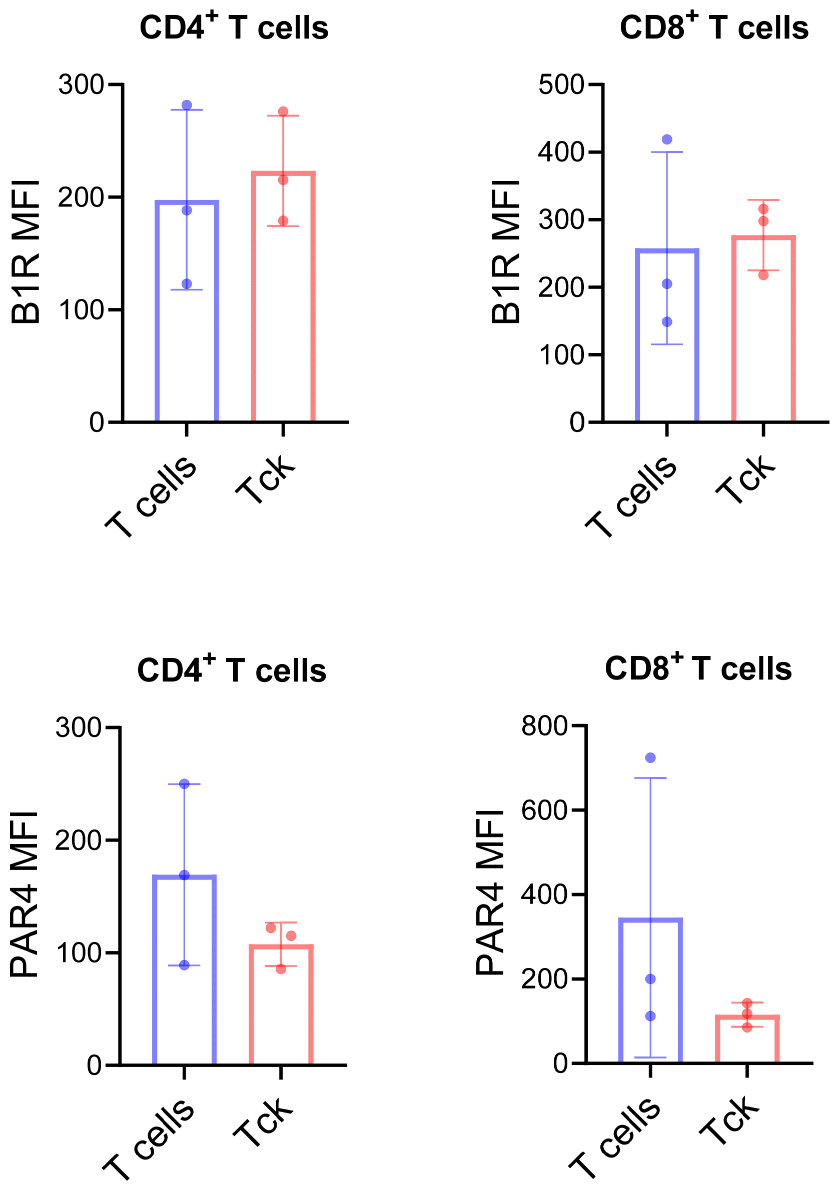

Supplemental Table 1. Relationships between sCD13 levels, gender, and patient comorbidities

	Comorbidities																			
	Diabetes		Heart disease		Renal disease		Lung disease		Autoimmune		Cancer		Obesity		Hypertension		Immune deficiency		History of smoking	
	-	+	-	+	-	+	-	+	-	+	-	+	-	+	-	+	-	+	-	+
All†	979 (1202)	932 (832)	976 (1002)	923 (1204)	926 (1027)	1040 (1155)	1030 (1173)	773 (677)	967 (1080)	834 (779)	936 (1037)	1115 (1244)	935 (989)	984 (1139)	771 (716)	1049 (1186)	936 (1058)	1047 (1197)	908 (1005)	1106 (1218)
Male†	1037 (1250)	1178 (1026)	1122 (1212)	1055 (1094)	1090 (1270)	1113 (990)	1180 (1255)	749 (562)	1113 (1180)	646 (624)	1112 (1204)	940 (898)	981 (1235)	1238 (1080)	1064 (1277)	1112 (1133)	1089 (1150)	1210 (1425)	1242 (1248)	826 (956)
Female†	1000 (1090)	519 (472)	903 (970)	452 (541)	845 (958)	539 (538)	720 (735)	889 (1116)	783 (913)	804 (737)	774 (942)	548 (586)	741 (1046)	817 (774)	905 (864)	710 (918)	798 (909)	546 (662)	718 (696)	1136 (1595)
P-value (Male vs. Female)	0.75	**0.001**	0.32	0.056	0.41	0.064	0.068	0.83	0.072	0.76	0.062	0.87	0.34	0.076	0.71	0.055	0.11	0.53	**0.01**	0.79

† sCD13 levels ng/ml: mean (standard deviation)

Supplemental Table 2. Relationship between sCD13 levels, race, and patient comorbidities

	Comorbidities																			
	Diabetes		Heart disease		Renal disease		Lung disease		Autoimmune		Cancer		Obesity		Hypertension		Immune deficiency		History of smoking	
	-	+	-	+	-	+	-	+	-	+	-	+	-	+	-	+	-	+	-	+
All†	979 (1202)	932 (832)	976 (1002)	923 (1204)	926 (1027)	1040 (1155)	1030 (1173)	773 (677)	967 (1080)	834 (779)	936 (1037)	1115 (1244)	935 (989)	984 (1139)	771 (716)	1049 (1186)	936 (1058)	1047 (1197)	908 (1005)	1106 (1218)
Black†	1336 (1453)	1101 (1029)	1285 (1332)	1142 (1247)	1330 (1429)	1086 (1031)	1382 (1351)	900 (1125)	1248 (1332)	1188 (570)	1223 (1350)	1405 (888)	1252 (1611)	1239 (1048)	1259 (1480)	1225 (1038)	1216 (1291)	1577 (1510)	1215 (1266)	1324 (1423)
White†	807 (829)	733 (729)	804 (832)	686 (610)	796 (824)	737 (707)	727 (795)	930 (774)	807 (800)	402 (547)	818 (848)	605 (406)	753 (757)	819 (845)	877 (737)	729 (118)	814 (805)	401 (479)	847 (817)	614 (707)
P-value (Black vs. White)	0.083	0.099	**0.02**	0.60	**0.03**	0.23	**0.003**	0.66	**0.045**	0.07	0.07	**0.03**	0.17	0.07	0.52	**0.01**	0.053	0.10	0.15	**0.02**

† sCD13 levels ng/ml: mean (standard deviation)

Supplemental Table 3. Subject characteristics of the Weill Cornel Medical College cohort

	COVID-19		Controls	
**Demographics**				
Number	21		4	
Age (years)*	62 ± 15	(29-85)	43 ± 19	(25-60)
Female	5	(23.8%)	3	(75.0%)
**Clinical Progression**				
WHO score	6 ± 2	(3-8)		
Mild/Moderate/Severe	2/9/10			
**Comorbidities**				
Diabetes	11	(52.4%)		
Hyperlipidemia	11	(52.4%)		
Renal disease	8	(38.1%)		
Lung disease	5	(23.8%)		
Hypertension	12	(57.1%)		
**Medications**				
Hydroxychloroquine	2	(9.5%)		
Anti-IL6 receptor	1	(4.8%)		
Steroids	17	(81.0%)		
Antiviral	10	(47.6%)		
**Respiratory status**				
Room air	2	(9.5%)		
Nasal cannula	8	(38.1%)		
High flow oxygen	3	(14.3%)		
Mechanical ventilation	8	(38.1%)		
**Final outcomes**				
Discharged	13	(61.9%)		
Death	8	(38.1%)		

* Mean ± standard deviation (range or percentage)

Supplemental Table 4. Subject characteristics of patients with sepsis

Characteristics	Sepsis group (n = 50)
**Age, mean (SD)**	52 ± 19.3
**Male, n (%)**	32 (64)
**Race, n (%)**	
White	43 (86)
Black	7 (14)
**Ethnicity, n (%)**	
Non-Hispanic or Latino	46 (92)
Hispanic or Latino	4 (8)
**Chronic comorbidities, n (%)**	
Cardiovascular disease	19 (38)
Pulmonary disease	19 (38)
Renal disease	20 (40)
Liver disease	15 (30)
Diabetes	14 (28)
Malignancy	11 (22)
**Mechanical ventilation, n (%)**	35 (70)
**SOFA score, mean (SD)**	6.4 ± (3.7)
**Hospital mortality, n (%)**	13 (26)
**Source of sepsis, n (%)**	
Pulmonary	25 (50)
Intra-abdominal	18 (36)
Skin soft tissue	4 (8)
Urinary	2 (4)
ENT	2 (4)
CNS	2 (4)
Pleural	1 (2)
Blood	2 (4)

Supplemental Table 5. List of neutrophil-immaturity genes

Neutrophil-immaturity genes	AZU1; BPI; CEACAM8; CTSG; DEFA1; DEFA1B; DEFA3; DEFA4; ELANE; LCN2; MPO; MS4A3; SLC2A5; CAMP; CD24; CD24L4; CEACAM6; CYP4F22; LTF; OLFM4; OLR1; PI3 RETN; SLPI; TNC1; EEF1E1; HP; HPR; IGLL1; KIAA0101; KIF11; MMP8; NKG7; PRG2; RNASE3; TARP; TMEM106C; TRGC1; TRGV9
